# High‐Resolution Multispectral Photovoltaic Imagers from Visible to Short‐Wave Infrared

**DOI:** 10.1002/advs.202519991

**Published:** 2026-01-20

**Authors:** Wanqing Li, Cheng Bi, Min He, Xiaolong Zheng, Yuning Luo, Yimei Tan, Chenxi Liu, Salihuojia Talanti, Yanfei Liu, Ge Mu, Qun Hao, Kangkang Weng, Xin Tang

**Affiliations:** ^1^ School of Optics and Photonics Beijing Institute of Technology Beijing China; ^2^ Yangtze Delta Region Academy of Beijing Institute of Technology Jiaxing China; ^3^ XinIR Technology (Beijing) Co., LTD Beijing China; ^4^ School of Optoelectronic Engineering Changchun University of Science and Technology Jilin China; ^5^ College of Science Minzu University of China Beijing China

**Keywords:** CMOS integration, multispectral imagers, organic photodetector, super‐resolution reconstruction

## Abstract

Visible to short‐wave infrared multispectral imaging is gaining significant attention across various fields, including agriculture, security, and medical diagnostics. Traditional multispectral imaging systems often rely on separate sensors for different spectral bands, leading to complex optical alignment and irreversible resolution loss. Here, we present hardware‐algorithm co‐designed architecture to achieve multispectral super‐resolution imaging. Specifically, we demonstrate a monolithic quad‐spectral photovoltaic imaging platform featuring a resolution of 640 × 512 pixels with <1% dead pixels per channel. The system achieves broadband spectral integration from visible to short‐wave infrared (350–2350 nm) by combining an all‐polymer bulk heterojunction with colloidal quantum dots within a single CMOS‐compatible architecture. The compatibility of all‐polymer bulk heterojunction with direct photopatterning allows for precise patterning and high‐density integration, enabling the devices to operate efficiently in photovoltage mode. To address resolution degradation inherent in planar‐integrated spectral sensing architectures, we applied a super‐resolution reconstruction method, restoring images to a resolution of 640 × 512. The demonstrated capability to simultaneously capture and process multispectral data paves the way for CMOS integration, multispectral Imagers, organic photodetector, super‐resolution reconstruction applications in diverse fields, from precision agriculture to medical diagnostics and beyond.

## Graphical abstract

We demonstrate a monolithic quad‐spectral imager that seamlessly integrates visible and short‐wave infrared detection on a single chip. Through direct photopatterning of an all‐polymer bulk heterojunction and colloidal quantum dots, the device achieves high‐resolution (640 × 512) imaging across 350–2400 nm, enabling multispectral capture for applications from precision agriculture to medical diagnostics.

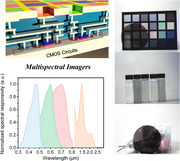

## 1 Introduction

Multispectral imaging spanning visible (Vis) to short‐wave infrared (SWIR) (350–2500 nm) plays vital roles in precision agriculture, medical diagnostics, and machine vision [1, 2, 3, 4, 5, 6]. However, conventional systems employing discrete silicon (Vis) and III–V semiconductor (SWIR) sensors face fundamental limitations [7]: (i) limited intrinsic spectral discrimination without optical filters, (ii) lack of universal nanofabrication protocols for heterogeneous photodetector arrays, and (iii) irreversible resolution loss in planar‐integrated spectral sensing architectures.

Biological vision's hierarchical spectral processing, where retinal L/M/S cones transduce wavelength‐specific photons into neural signals refined across V1–V4 cortical stages. Inspired by this natural architecture, a multispectral high‐resolution imaging system could be developed by integrating spectrally selective optoelectronic arrays with neuromorphic processing cores. For wavelength‐specific optoelectronic materials, solution‐processed semiconductors—organics, perovskites, and colloidal quantum dots (CQDs)—offer promising alternatives [8, 9, 10, 11]. In particular, organic photodetectors demonstrate excellent performance in visible detection with intrinsic spectral selectivity [12, 13]. Meanwhile, CQDs offer size‐tunable bandgaps that enable spectral detection extending into the short‐wave infrared and even long‐wave infrared regions [14, 15, 16]. Thus, integrating organic semiconductors with CQDs on a single chip offers a promising route toward fabricating filter‐free, broadband multispectral image sensors.

High‐density integration with CMOS‐compatible architectures requires photodetection units that are amenable to array patterning and capable of operating at low voltages. Due to the high exciton binding energies of organic semiconductors [17, 18], photodetectors based on single‐component organic systems typically exhibit poor low‐bias performance. It often necessitates large operating voltages that are incompatible with CMOS integration [19]. High‐performance organic devices generally employ bulk heterojunction (BHJ) structures, where the built‐in potential enables efficient operation at zero or low bias by leveraging interfacial energetics between donor and acceptor materials [20]. Nevertheless, patterning BHJ structures remains a significant challenge: such systems require simultaneous stabilization of both donor and acceptor phases during processing. Inkjet/screen printing patterning methods [21, 22, 23] struggle to simultaneously achieve <10 µm feature resolution and high‐throughput manufacturing while maintaining the critical phase purity. Conventional photoresist‐based methods often induce solvent erosion, compromising material integrity. Although direct photopatterning has been successfully applied to single‐component systems [24, 25, 26, 27], it encounters significant challenges in multiphase BHJs. The mismatch in photo‐crosslinking kinetics between different molecular components typically results in poorly defined patterns and morphological degradation. This underscores the necessity for precise matching of the photo‐crosslinking kinetics between donor and acceptor components to suppress phase segregation, which is a critical requirement that remains unaddressed in prior research [28].

**FIGURE 1:**
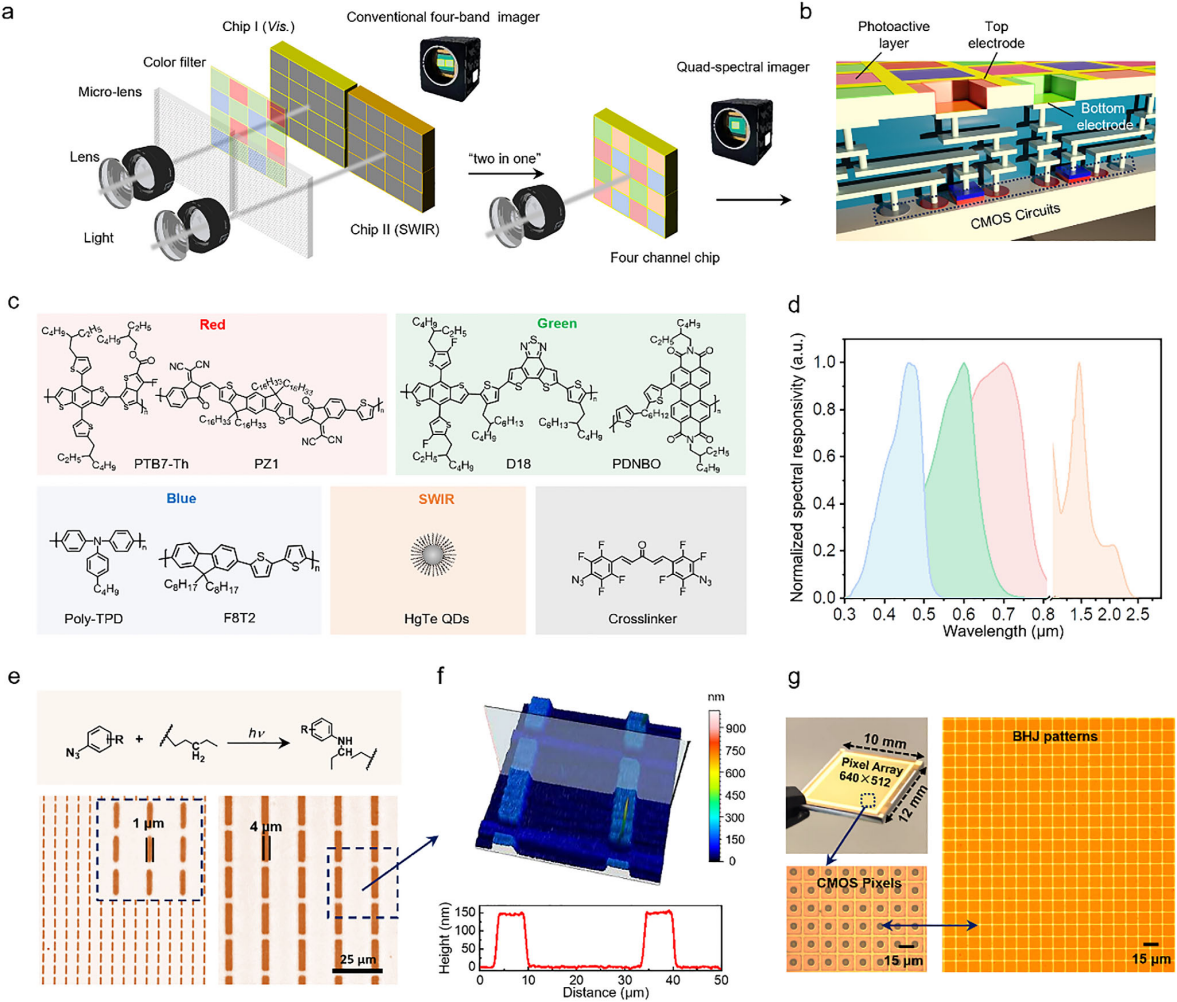
Design of RGB‐SWIR multispectral FPA imagers. a) Schematic illustration comparing the typical two‐chip design (left) with the integrated “two‐in‐one” design (right) for visible and SWIR quad‐spectral imaging sensors. b) Schematic diagram of the four‐channel chip used in this study, highlighting the integration of blue, green, red, and SWIR photodetectors on a single ROIC. c) Chemical structures of the materials employed in the quad‐spectral imaging sensor, including the polymer‐based bulk heterojunctions and HgTe CQDs. d) Responsivity spectra of the blue, green, red, and SWIR photodetectors. e) Top: Schematic of the C‐H insertion reaction used for patterning polymer BHJs and CQDs. Bottom: Optical microscope image of patterned PTB7‐Th/PZ1 red BHJs. f) Top: 3D morphology of the BHJ patterns. Bottom: Profile curves of the BHJ patterns. g) Top: Photograph of the CMOS ROICs with a resolution of 640 × 512 pixels. Bottom: Optical microscope images of organic patterns tailored to match the pixel dimensions of the CMOS ROICs.

Here, we demonstrate a direct photopatterning strategy for monolithic integration of coaxial visible‐to‐SWIR (350–2350 nm) quad‐spectral imager, combining all‐polymer BHJs and HgTe CQDs into a 640 × 512 focal plane array, this approach avoids the complex beam‐splitting architectures and separate optical paths found in conventional visible‐to‐SWIR imagers (Figure 1a). Kinetic matching of photo‐crosslinking rates between BHJ constituents ensures uniform stabilization. With less than 1% dead pixels per channel, the multispectral imagers show good reliability and imaging quality. To overcome the spatial resolution constraints inherent in planar‐integrated spectral sensing architectures, we developed a super‐resolution reconstruction framework specifically for quad‐channel spectral imaging chips. This framework incorporates a feature enhancement network that synergistically integrates channel‐wise and spatial attention mechanisms. In contrast to conventional micro‐electro‐mechanical systems (MEMS) fabrication techniques relying on microlens arrays and Bayer filter arrays, our approach achieves monolithic integration of multi‐spectral detection units via direct‐write photolithographic patterning. This effectively circumvents the longstanding compatibility challenges between infrared readout integrated circuits (ROIC) with larger pixel pitches and standard visible‐light CMOS fabrication processes. Experimental validation demonstrates that the proposed system acquires high‐fidelity multispectral image sets with enhanced spatial resolution, validating its capability to preserve spectral‐spatial coherence while addressing the inherent trade‐offs in compact multispectral sensor design.

## 2 Results

### 2.1 Fabrication of RGB‐SWIR Multispectral Photodetector Arrays

Figure 1b illustrates the schematic architecture of the RGB‐SWIR quad‐channel photodetector chip. Each channel is tailored for a distinct spectral segment, encompassing blue, green, red, and SWIR detection. The visible spectrum channels employ all‐polymer BHJs, which enable precise wavelength targeting. Specifically, the photoactive polymer BHJ layer utilizes poly(4‐butylphenyldiphenylamine) (Poly‐TPD) and poly(9,9‐dioctylfluorene‐alt‐bithiophene) (F8T2) for blue‐selective photodetection; poly[(2,6‐(4,8‐bis(5‐(2‐ethylhexyl‐3‐fluoro)thiophen‐2‐yl)‐benzo[1,2‐b:4,5‐b']dithiophene))‐alt‐5,5'‐(5,8‐bis(4‐(2‐butyloctyl)thiophen‐2‐yl)dithieno[3',2':3,4;2'',3'':5,6]benzo[1,2‐c][1,2,5]thiadiazole)] (D18) and poly(dithieno[3,2‐b:2',3'‐d]silole‐alt‐naphtho[1,2‐b:5,6‐b']dithiophene) (PDNBO) for green‐selective photodetection; and poly[4,8‐bis(5‐(2‐ethylhexyl)thiophen‐2‐yl)benzo[1,2‐b:4,5‐b']dithiophene‐2,6‐diyl‐alt‐(4‐(2‐ethylhexyl)‐3‐fluorothieno[3,4‐b]thiophene‐2‐carboxylate‐2‐6‐diyl)] (PTB7‐Th) with 2‐((Z)‐2‐((7‐(((Z)‐3‐(dicyanomethylene)‐5‐(5‐methylthiophen‐2‐yl)‐1‐oxo‐1,3‐dihydro‐2H‐inden‐2‐ylidene)methyl)‐4,4,9,9‐tetrahexadecyl‐4,9‐dihydro‐s‐indaceno[1,2‐b:5,6‐b']dithiophen‐2‐yl)methylene)‐6‐methyl‐3‐oxo‐2,3‐dihydro‐1H‐inden‐1‐ylidene)malononitrile (PZ1) for red‐selective photodetection (Figure 1c). As shown in Figure S1a–f, the donor and acceptor materials within each color channel exhibit closely matched absorption profiles. This spectral overlap ensures a consistent spectral response across the active layer, which is critical for accurate color detection.

To extend the spectral response into the SWIR region, HgTe CQDs with diameters around 4.1 nm were integrated into the detector design (Figure S2). The HgTe CQDs exhibit strong absorption across the SWIR spectrum, with a cut‐off edge of 2350 nm (Figure S1g). This design ensures that the photodetectors can accurately differentiate colors across the visible‐SWIR spectrum. As illustrated in Figure 1d, the photodetectors exhibit color‐selective responsivity across the visible and SWIR regions. The normalized responsivity spectra display distinct peaks at 460, 600, 700, and 1470 nm, respectively. This spectral selectivity enables efficient and accurate detection within the visible and infrared ranges. The sharp and well‐separated peaks ensure minimal cross‐talk between channels, enhancing the overall performance and reliability of the multispectral photodetector.

We developed a direct photopatterning process utilizing a C‐H insertion reaction with (1E,4E)‐1,5‐bis(4‐azido‐2,3,5,6‐tetrafluorophenyl) penta‐1,4‐dien‐3‐one as the crosslinker. Note that material systems with kinetically matched photo‐crosslinking rates were used for their good compatibility in dynamic crosslink processes. For the visible light detection, we applied all‐polymer BHJ materials. For the SWIR detection, we applied HgTe CQDs. The photopatterning process consists of three primary steps (Figure S3): (i) Ink application: An ink composed of BHJ material mixed with bisazide additives is applied onto the silicon ROICs. (ii) UV exposure: The layers are exposed to ultraviolet light (365 nm) through a photomask, initiating a photochemical reaction. This UV exposure triggers the release of nitrogen from the bisazides, generating highly reactive nitrene radicals. The nitrene radicals form covalent bonds with the long alkyl chains of the polymers or CQD ligands via C‐H insertion (Figure 1e, top). This reaction results in a crosslinked, insoluble network within the exposed areas of the film. (iii) Development: Subsequent development with a chlorobenzene removes the unexposed BHJ materials, leaving behind a patterned, functional layer. By sequentially applying different BHJs tailored to specific wavelengths, multi‐material stacks arrays can be achieved.

The achievement of matched photo‐crosslinking kinetics between all‐polymer BHJ components enables direct photopatterning of BHJs, achieving <5 µm feature resolution critical for multispectral pixel integration. This kinetic compatibility ensures simultaneous curing of dissimilar materials while preserving BHJ components integrity. Patterned stripes of B‐, G‐, and R‐BHJs, as well as SWIR CQDs, are depicted in various configurations such as microdots, diamonds, triangles, letters, and lines in Figure 1e and Figures S4–S8. These polymer BHJ patterns achieve a minimum line width of 1 µm, matching the resolution limits of the predesigned photomask. The height profile of the line patterns (Figure 1f) demonstrates the high uniformity of these patterns, with a film thickness of approximately 150 nm and sharp edges. The film thickness can be precisely controlled by adjusting the concentration of the ink and the coating parameters. A thickness of 150 nm is sufficient for fabricating high‐performance organic photodetectors. Furthermore, BHJ layers can be monolithically integrated with ROICs. The CMOS chip features a 640 × 512‐pixel array with a 15 µm pitch, and the BHJ layers are patterned to match this layout exactly (Figure 1g). This integration ensures precise alignment and optimal performance, making it ideal for high‐resolution imaging applications.

Atomic force microscopy (AFM) images of the pristine and patterned films exhibit a similar surface roughness (Figure S9). This indicates that the patterning process minimally changes the BHJ and HgTe CQD morphology, preserving the original material properties. Scanning electron microscopy (SEM) images (Figure S10a) reveal a marked contrast between the CQD pattern and substrate. The line patterns also show the high uniformity with sharp edges (Figure S10b). Top‐view SEM images (Figure S10c,d) show patterned HgTe CQD films with densely packed, uniform CQDs, demonstrating the effectiveness of the photopatterning method. The ability to pattern HgTe CQD and BHJ films while preserving their morphological and compositional integrity ensures high‐performance and reliable devices, suitable for a wide range of applications.

### 2.2 Properties of Patterned Photodetectors

**FIGURE 2:**
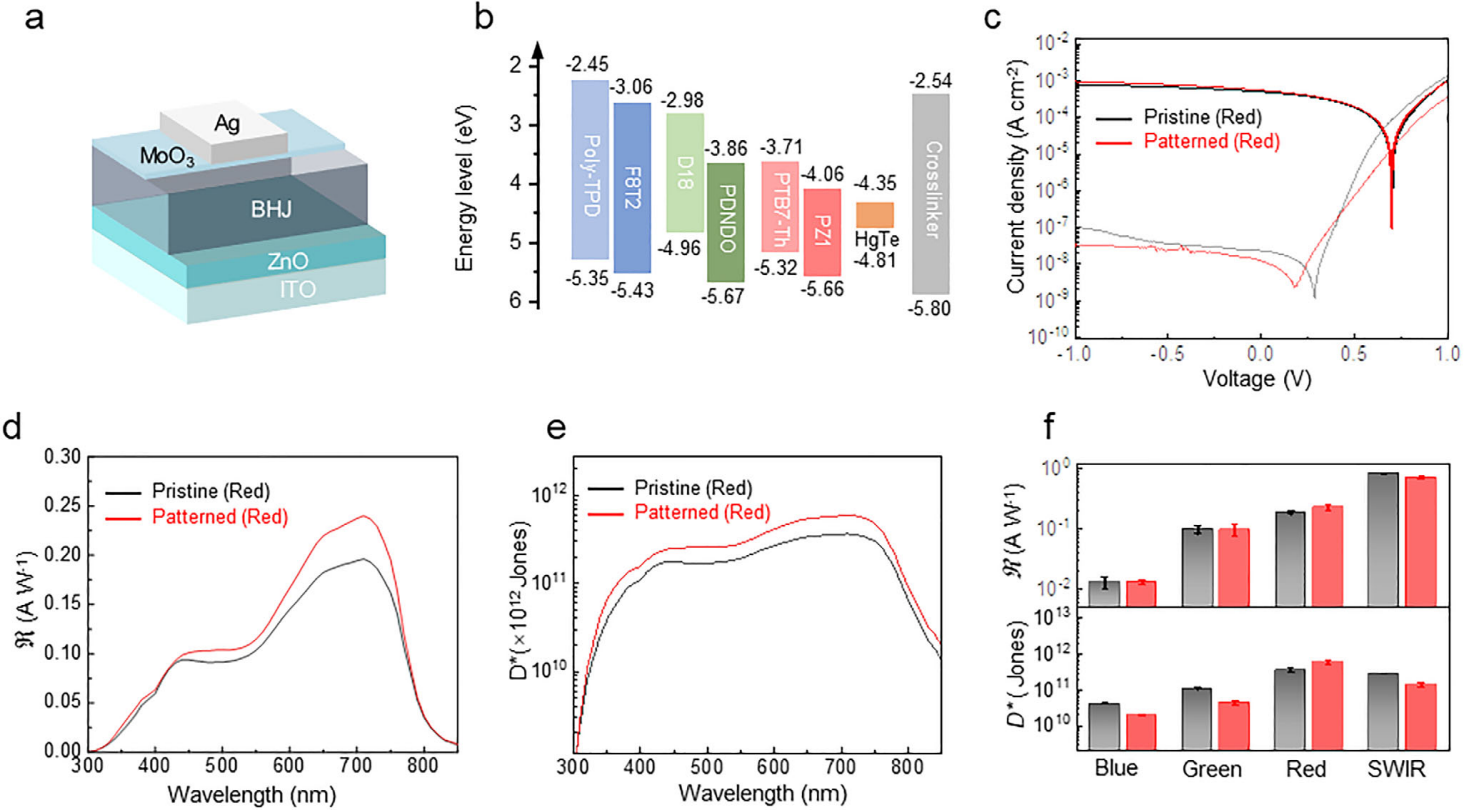
Patterned single‐element devices performance. a) The device structure of polymer photodetector. Dark current density: semi‐transparent curves. Photocurrent density: solid curves. b) Energy level diagram of materials used to fabricate four‐spectrum photodetectors. c) *J* – *V* curves of pristine and patterned red‐detector in dark and under illumination. d) Spectral responsivity of pristine and patterned red‐detectors. e) Detectivity spectrum of pristine and patterned red‐detectors. f) *ℜ* and D* of BHJ detectors (for B, G, R) and HgTe CQDs (for SWIR) after patterning process. Black box: pristine detectors. Red box: patterned detectors.

Nondestructive patterning methods are critical for maintaining the optoelectronic integrity of high‐performance photodetectors. To evaluate pixel‐patterning compatibility in photodiode architectures, we fabricated single‐element devices employing organic BHJs and HgTe CQDs active layers. For organic BHJ‐based PDs, the inverted device architecture comprises sequentially deposited layers: ITO/ ZnO/ BHJ / MoO_3_/ Ag (Figure 2a). Figure 2b presents the schematic energy level diagram of all photoactive layer compositions and the crosslinker used in patterned devices. The optimized energy level offset between the BHJ and HgTe (HOMO/LUMO or VB/CB levels detailed in Figure S11 and Table S1).

Optoelectronic characterization was performed through current density‐voltage (*J–V*) analysis, EQE measurements, spectral responsivity, detectivity, and temporal response evaluation. Figure 2c compares the *J–V* characteristics of pristine and patterned devices under 660‐nm illumination (1 mW cm^−2^) and dark conditions, with a focus on red PDs for clarity. Patterned red‐PDs exhibit a dark current density (*J_d_*) of 1.2 × 10^−8^ A cm^−2^ at 0 V bias, lower than the 2.3 × 10^−8^ A cm^−2^ in pristine devices, suggesting the patterning process can reduce leakage pathways from optimized interfacial engineering [29, 30, 31]. Nearly identical photocurrent density‐voltage (*J*_ph_‐*V*) curves between pristine and patterned devices confirm the low‐damage nature of the photopatterning process. Zero‐bias EQE measurements (Figure S12) reveal a peak value (650 nm) of 43% for patterned red‐PDs, surpassing the 35% achieved by pristine counterparts.

Figure 2d displays the responsivity (*R*, units of A W^−^^1^) of red photodetectors calculated from the equation

(1) $$R=\mathrm{EQE}\cdot \frac{e}{hv}$$

where *e* is the elementary charge, *h* is Planck's constant, and *v* is the frequency of light. The pristine red PDs demonstrate a peak responsivity of 0.2 A W^−1^ under zero‐bias conditions. The patterned PDs exhibit enhanced performance in the red spectral regime, achieving a peak responsivity of 0.24 A W^−1^ at zero bias.

The root‐mean‐square (RMS) noise current is also measured (Figure S13). The RMS noise current of pristine and patterned devices is 0.09 and 0.12 pA, respectively. The detectivity (D*, units of Jones) of photodetectors is determined by the equation:

(2) $$D^{*}=\frac{\sqrt{A}}{I_{n}}\;R\;$$

where *A* is the sensing area, *I_n_* is the measured RMS noise, and *R* is the responsivity. Figure 2e shows the specific detectivity spectra of the red PD under zero bias, with peak values of 3.7 × 10^11^ Jones for the pristine PD and 6 × 10^11^ Jones for the patterned PD. Similarly, the pristine and patterned blue and green photodetectors demonstrate comparable performance metrics. As illustrated in Figures S14 and S15, patterning process maintains critical parameters including dark current density, responsivity, and detectivity. Although the current device parameters are lower than that of state‐of‐the‐art organic BHJ devices, further refinement of the material system (e.g., interfacial engineering, phase‐separation control) is expected to bridge this efficiency discrepancy.

The dynamic response characteristics of photodetectors were quantified through rise/fall time analysis under zero‐bias operation. Figure S16 displays the voltage temporal response of red, green, and blue PDs under pulsed LED illumination. The patterned blue, green, red PD shows a fast *τ_raise_* of 54.6, 72.7, 66.2 µs and *τ_drop_* of 118.4, 99.5, 242.2 µs, respectively. *τ_raise_* is time for photovoltage increases from 10% to 90% of the peak value and *τ_raise_* is time for photovoltage decreases from 90% to 10% of the peak value.

The SWIR PDs performance was characterized using a calibrated 600°C blackbody radiation source under controlled illumination conditions (Figure S17a). The device structure consists of stacked layers: ITO/ZnO/HgTe CQDs/P3HT/MoO_3_/Ag (Figure S17b). For HgTe CQD films, a critical optimization step involves treating the layer with a short‐chain ethanedithiol (EDT) solution. This process facilitates the transition from the initial long‐chain dodecanethiol (DDT) capping ligands to EDT, which is central to enhancing the film's electronic and structural properties, as supported by the Fourier Transform infrared spectroscopy (FTIR) data presented in Figure S18. The distinction from Vis‐PDs is the implementation of a P3HT electron‐blocking layer, which serves dual functions: enhancing device performance through carrier management and enabling photopatterning compatibility between SWIR‐PDs and Vis‐PDs [32]. Cross‐sectional TEM images of the pristine and patterned SWIR detector are presented in Figure S17c,d, respectively. Figure S17e demonstrates the *J–V* characteristics under dark and illuminated conditions. At 0 V bias, *J_d_* measure 6.2 × 10^−7^ A cm^−2^ and 5.61 × 10^−7^ A cm^−2^ for pristine and patterned devices, respectively. Under blackbody illumination, *J*_ph_ reach 6.82 × 10^−4^ A cm^−2^ (pristine) and 4.55 × 10^−4^ A cm^−2^ (patterned). EQE measurements reveal good carrier collection efficiency, with pristine devices achieving 72.3% EQE at 1490 nm under zero bias, maintaining spectral response up to 2.4 µm cutoff wavelength (Figure S17f). Patterned counterparts exhibit 62.1% EQE at 1460 nm with identical spectral extension. This high EQE originates from optimized band alignment across the multilayer stack, facilitating efficient carrier separation and transport [29]. The fabricated devices exhibit peak responsivities of 0.87 and 0.73 A W^−1^ for the pristine (1500 nm) and patterned (1470 nm) configurations, respectively (Figure S17g). RMS noise under zero bias present 0.67 and 1.1 pA for the pristine and patterned devices, respectively. Specific detectivity spectra under zero bias (Figure S17h) demonstrate peak values of 2.91 × 10^11^ Jones and 1.43 × 10^11^ Jones for pristine and patterned devices, respectively. To better understand the low‐frequency noise characteristics of the devices, noise spectral density measurements were performed on all four devices using a noise spectrum analyzer. The results are presented in Figure S19 and summarized in Table S2. As shown in Figure 2f, the *R* and D^*^ of both RGB and SWIR photodetectors show no significant variation before and after direct photopatterning, indicating that the all‐polymer BHJ and CQD‐based system are highly suitable for fabricating nondestructive multispectral photovoltaic detector arrays.

Note that, for HgTe CQDs, the film retention rate after direct photopatterning is relatively low, and a single‐layer photopatterned film typically does not provide sufficient thickness to ensure adequate SWIR light absorption. To overcome this limitation, we adopted a multi‐step photopatterning process, repeating the patterning cycle five times. However, such repeated lithography steps increase processing complexity and may introduce excessive impurities, which can compromise film uniformity and ultimately degrade the imaging performance when integrated into a focal plane array (FPA). Therefore, future device optimization should focus on developing deposition techniques capable of achieving thicker single‐layer films in a single lithography step.

### 2.3 RGB‐SWIR Multispectral FPA Imagers

**FIGURE 3:**
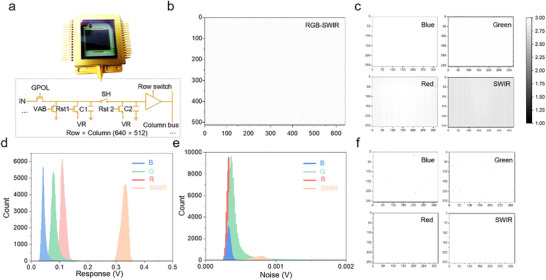
Performance characteristics of the RGB‐SWIR multispectral FPA imagers. a) Schematic diagram illustrating the architectural configuration of the quad‐spectral imaging chips and corresponding electronic circuitry of chip‐integrated pixels. b) The dark‐current mapping of the quad‐spectral FPA imager. c) Channel‐specific dark current mapping images (256 × 320 resolution) obtained through spectral decomposition for individual red, green, blue, and SWIR detection channels. d) Statistical histograms quantifying pixel response uniformity across different spectral channels in the FPA imager. e) Distribution histogram of the noise of each channel pixel in the quad‐spectral FPA imager. f) Channel‐specific dead pixel mapping images (256 × 320 resolution) for individual red, green, blue, and SWIR detection channels. The color bar represents the chip output voltage, ranging from ∼3 V (baseline ROIC output without any photoactive material) to ∼1 V (full‐well output under maximum signal).

The proposed direct photopatterning method demonstrates full compatibility with standard CMOS fabrication processes. We monolithically integrated three polymer‐BHJ‐based PDs with HgTe CQD‐based SWIR photodetector onto a single ROIC. This hybrid integration strategy enables the realization of a multispectral FPA imager based on a 640 × 512 pixel ROIC with 15 µm pitch, where the RGB and SWIR detectors are arranged in a 2 × 2 pixel pattern, resulting in effective 320 × 256 resolution per subarrays. Figure 3a shows the architecture of the multispectral chips and associated electric circuit of pixels. The four spectral channels were spatially partitioned into 320 × 256 subarrays through direct photopatterning, with their geometric arrangement detailed in Figure S20. Figure 3b presents the full‐array dark current distribution (640 × 512 pixels), exhibiting a median dark current of 0.19 pA under 2 V bias across all spectral bands. Post‐demultiplexing dark current distributions for individual RGB and SWIR channels are shown in Figure 3c, revealing channel‐specific dark current characteristics of 0.06, 0.08, 0.14, and 0.46 pA for blue, green, red, and SWIR detectors, respectively.

Photoresponse uniformity was evaluated under tungsten‐halogen illumination (Figure S21). Figure 3d quantifies the response voltage distribution through Gaussian‐fitted histograms, showing peak response voltages of 41.9, 77.8, 109, and 331.8 mV for blue, green, red, and SWIR channels at tungsten‐halogen illumination. Noise characterization with post‐demultiplexing array noise distribution profiles provided in Figure 3e. Post‐processing defect analysis revealed that the dead pixel rates across spectral channels remained below 1%, as illustrated in the demultiplexed dead pixel maps (Figure 3f). Detailed performance parameter extraction methodology is documented in Section S4.

### 2.4 Multispectral Imaging and Super‐Resolution Reconstruction Using Multispectral Imager

The multispectral imager architecture, while enabling multispectral (RGB‐SWIR) detection through spatial partitioning of photodetector materials, inherently reduces the native resolution of each spectral channel to 256 × 320 pixels—a critical trade‐off arising from the fourfold pixel allocation. To overcome this resolution limitation, we propose a super‐resolution reconstruction method for quad‐channel spectral imaging chips, constructing a feature enhancement network integrating channel‐spatial attention mechanisms [33, 34]. At the hardware level, we employ photolithography to directly fabricate 2 × 2 periodically arranged spectral sensing unit arrays within CMOS pixel regions. Through heterogeneous integration of photosensitive materials, each unit achieves synchronous capture of four characteristic spectral bands spanning 350–2350 nm. Compared with traditional MEMS manufacturing processes using microlens arrays, this scheme realizes multi‐spectral detection unit integration through direct‐write lithography, effectively resolving the compatibility issues between infrared readout circuits and visible‐light CMOS processes. The detail of super‐resolution reconstruction method was presented in Section S4.

**FIGURE 4:**
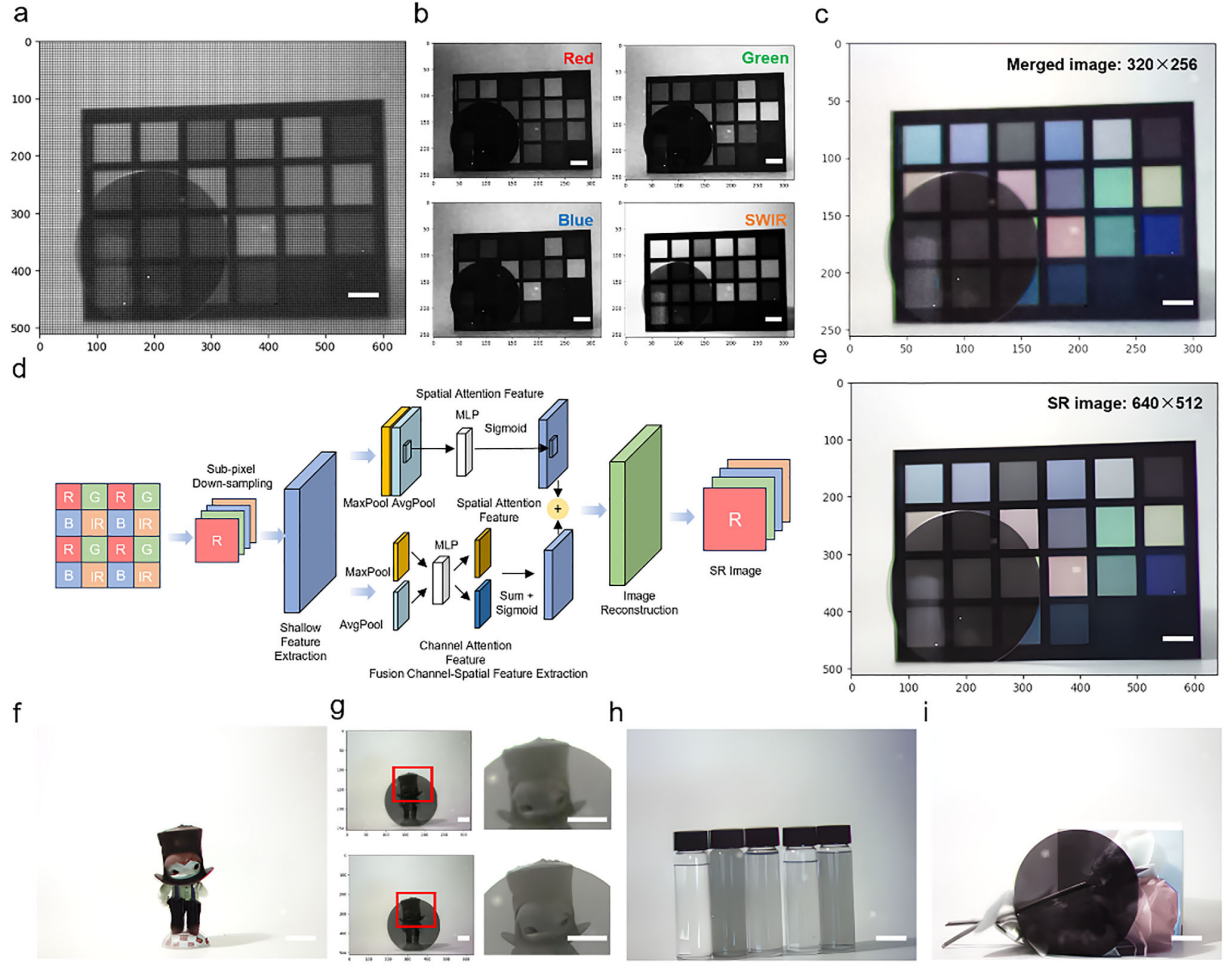
Quad‐spectral imaging and super‐resolution reconstruction using multispectral imager. a) Raw 640 × 512 image captured by the quad‐spectral imager, featuring a 24‐color calibration chart, silicon wafer. b) Decomposed 256×320 monochromatic images for red, green, blue, and SWIR channels. c) Fused quad‐spectral (RGB + SWIR) composite image at 256 × 320 resolution. d) Schematic diagram of the hybrid algorithm pipeline for spectral fusion and super‐resolution reconstruction. e) 640 × 512 super‐resolved RGB‐SWIR composite image. f) Demonstration of super‐resolution enhancement on a cartoon character (640 × 512). g) Magnified comparison of a representative region (red box) pre‐ and post‐super‐resolution processing. h) SWIR‐dominant imaging of glass vials containing organic solvents (tetrachloroethylene, water, dodecanethiol, isopropanol, and ethanol, left to right). i) Multispectral floral imaging. Scale bars: 2 cm.

Figure 4a captures the raw quad‐spectral image of a 24‐color calibration chart and silicon wafer. Post‐demultiplexed subarrays (Figure 4b) exhibit channel‐specific resolution constraints, particularly in the SWIR channel where 15 µm pixel pitch limits feature discernibility. Figure 4c shows the merged image where the resolution degradation is evident in the pixelated edges of the color patches. This resolution compromise necessitated the implementation of three‐stage cascaded deep neural network architecture (Figure 4d): (i) A shallow feature extraction module employing 3 × 3 convolutional kernels for initial feature mapping; (ii) A deep feature enhancement module integrating dual channel‐spatial attention mechanisms, implementing cross‐spectral feature correlation modeling through cascaded channel attention submodule (FCSFSR‐C) and spatial attention submodule (FCSFSR‐S) (quantitative evaluation of FCSFSR was discussed in Section S5); (iii) An image reconstruction module utilizing sub‐pixel convolution for 2× upsampling. Notably, we propose a multimodal feature fusion strategy at the feature enhancement stage: Channel statistical features are extracted through global average pooling (GAP) and global maximum pooling (GMP), generating channel weight matrices via multilayer perceptron (MLP), while spatial attention mechanisms establish pixel position correlation matrices. Final synergistic optimization of channel‐spatial features is achieved through Hadamard product operations. The algorithm achieves a 2× resolution enhancement (Figure 4e,f), recovering critical details such as the sharp edge of the silicon wafer. Magnified comparisons (Figure 4g) reveal the algorithm's capacity to reconstruct photonically accurate edges in both visible and SWIR domains. The SWIR channel's resolution recovery enables multispectral discrimination: Figure 4h demonstrates the discrimination of five distinct solvents (tetrachloroethylene, water, dodecanethiol, isopropanol, and ethanol, arranged left to right) through their distinct SWIR absorption fingerprints. Flowers imaging (Figure 4i) further validates the system's quad‐color resolution capability. This resolution‐compensation strategy establishes a paradigm for multispectral systems, demonstrating that algorithmic recovery can transcend the traditional trade‐off between spectral channels and spatial resolution in monolithically integrated detectors.

## 3 Conclusions

In this work, we demonstrate hardware‐algorithm co‐designed architecture to achieve multispectral super‐resolution imaging, enabling the fabrication of a 640 × 512‐pixel multispectral imager spanning visible‐shortwave infrared (350–2350 nm) within a single CMOS‐compatible platform. The photo‐crosslinking kinetics of polymer donors and acceptors were precisely engineered to achieve <1 µm patterned features while preserving nanoscale BHJ morphology and device performance. We implemented a super‐resolution reconstruction method that leverages spectral correlation priors, restoring images to the native 640 × 512 resolution.

The monolithic integration of spectrally distinct, patterned BHJs and CQD pixels presents a specific set of advantages and trade‐offs compared to alternative multispectral imaging architectures. Hybrid systems employing MEMS filter wheels offer superior spectral tuning flexibility but introduce mechanical complexity, power consumption, and challenges in miniaturization [35]. Conversely, systems using printed color filter arrays on broadband detectors are highly cost‐effective but suffer from inherent optical losses and higher spectral crosstalk [36]. Our direct‐patterning approach occupies a middle ground, prioritizing structural compactness, good spatial registration, and passive, high‐speed operation, attributes critical for integrated SWIR imaging systems.

We recognize that although our PDs demonstrate competitive overall performance, certain metrics may fall short of the highest reported values for specific material systems (Tables S6 and S7). This stems from a deliberate design trade‐off: our material platform was selected primarily for its compatibility with monolithic, high‐density, patterned integration, a key enabler for scalable on‐chip fabrication. To advance performance further, future work will focus on developing novel polymer systems that retain excellent direct‐patterning capability while offering enhanced.

## 4 Methods

### 4.1 Materials

F8T2 was purchased from Luminescence Technology Co., Ltd; Poly‐TPD was purchased from Xi'an Yuri Solar Co. Ltd.; D18, PTB7‐Th, and PZ1 were purchased from Solarmer Materials Inc. Zinc acetate dihydrate, ethanolamine, 2‐methoxyethanol, isopropanol, MoO_3_, chlorobenzene, tellurium powder, oleylamine, trioctylphosphine, dodecanethiol, tetrachloroethylene, and ethanedithiol were purchased from Sigma–Aldrich. Crosslinker (1E,4E)‐1,5‐bis(4‐azido‐2,3,5,6‐tetrafluorophenyl)penta‐1,4‐dien‐3‐one was purchased from Dreamchem Inc. P3HT was purchased from 1‐Material Inc. HgCl_2_ (99%), were purchase from Strem Chemicals. All chemicals were used as received without further purification.

### 4.2 Single‐Element Device Fabrication

To fabricate the RGB photodetectors, the patterned ITO glass substrate was sequentially ultrasonicated in detergent, deionized water, and ethanol, each for 10 min and repeated twice, followed by drying with nitrogen gas. Subsequently, a ZnO sol‐gel solution, prepared by dissolving zinc acetate dihydrate (1 g), ethanolamine (0.28 g), and 2‐methoxyethanol (10 mL), was spin‐coated onto the cleaned ITO substrate at 2000 rpm for 20 s. The film was annealed at 200°C for 20 min to form a uniform ZnO layer. For the active layer PTB7‐Th: PZ1 (1:1, w/w), Poly‐TPD: F8T2 (1:1, w/w), and D18: PDNBO (1:1, w/w) were dissolved in chlorobenzene, with concentrations of 9 mg mL^−1^, 10 mg mL^−1^, and 7 mg mL^−1^, respectively. These solutions were spin‐coated onto the ZnO layer at 1000 rpm using a 0.45 µm filter and annealed in a nitrogen‐filled glove box at 100°C for 10 min. For patterned devices, 3% (w/w) photocrosslinker was added to each solution, which was then spin‐coated, irradiated under a 365 nm UV lamp (3 mW cm^−2^) for 1 min and developed with its original solvent, followed by annealing at 100°C for 10 min. Finally, MoO_3_ (3 nm)/Ag (100 nm) electrodes were deposited onto the active layers via thermal evaporation under high vacuum, with an active area of 0.05 cm^2^.

For the fabrication of SWIR photodetectors, the ITO cleaning, ZnO coating, and MoO_3_/Ag electrode deposition processes were similar to those used for the RGB PDs. For pristine SWIR PDs, HgTe CQDs (60 mg mL^−1^) were spin‐coated onto the ZnO film at 2000 rpm in five consecutive layers. Each layer was crosslinked with an ethylenediaminetetraacetic acid/isopropanol (EDT/IPA, 1:50 by volume) solution for 20 s, rinsed with IPA, and blow‐dried. For patterned SWIR PDs, the HgTe CQDs (60 mg mL^−1^) were spin‐coated onto the ZnO film at 1000 rpm was mixed with 20 wt.% crosslinker before spin‐coating. Each of the five layers was first photocrosslinked using the crosslinker, then treated with the EDT/IPA solution for 20 s, rinsed with IPA, and blow‐dried. A P3HT layer (mixed with 3% photocrosslinker, 10 mg mL^−1^ in CB) was spin‐coated onto the HgTe layer at 2500 rpm, irradiated under a 365 nm UV lamp (3 mW cm^−2^) for 1 min. Finally, MoO_3_ (3 nm)/Ag (100 nm) electrodes were deposited onto the active layers via thermal evaporation under high vacuum.

### 4.3 Multispectral FPA Imager Fabrication

The ROICs with a 640 × 512 pixel array and 15 µm pixel pitch were employed to fabricate FPA imagers. The fabrication process proceeded as follows: First, a ZnO electron‐transport layer was uniformly deposited on the ROIC substrate, following the same procedure used for the single‐element detectors. Subsequently, the SWIR (HgTe) pixel array was formed. In contrast to the single‐element detector, the HgTe layer on the FPA was deposited via spin‐coating (2000 rpm) using a solution of HgTe CQDs (30 mg mL^−^^1^) blended with 20% photocrosslinker. Laser direct writing (DWL66+, Heidelberg Instruments) was selectively performed on the SWIR channel regions. After development using chlorobenzene, patterned HgTe arrays were obtained. Then, patterned HgTe arrays treated with the EDT/IPA solution for 20 s, rinsed with IPA, and blow‐dried. This process was repeated five times to achieve a total HgTe array thickness of approximately 150 nm. Subsequently, red, blue and green channel arrays were fabricated using same methods. For each color channel (blue, green, and red), a single photopatterning cycle was conducted, with the same material composition and processing parameters as those employed for the single‐element device. After all color channels were defined, a P3HT hole‐transport layer was deposited over the SWIR channel and patterned via direct photolithography. Finally, top electrodes consisting of MoO_3_ (3 nm)/Ag (10 nm) were deposited onto the entire active area through thermal evaporation under high vacuum.

### 4.4 Characterization Techniques

The photodetector was characterized in a thermostat. For the spectral response characterization of the RGB photodetectors, light‐emitting diodes (LEDs) were used as the illumination sources. A 455 nm LED (M455D3, ThorLabs) was employed for the blue photodetectors, a 530 nm LED (M530L4, ThorLabs) for the green photodetectors, and a 660 nm LED (M660L4, ThorLabs) for the red photodetectors. Light intensity was verified with a Thorlabs SM1PD1B Si p–i‐n diode. UV–vis absorption spectra of BHJ films were measured with an Agilent Cary 5000 UV‐Vis‐NIR spectrophotometer. For SWIR photodetector measurements, the setup for spectral response measurements is illustrated in Figure S17a. A calibrated high‐temperature blackbody source, operating at 600°C, served as the light source for these measurements. The absorption spectra of HgTe CQD were characterized by a Fourier transform spectrometer (Nicolet iS20 FTIR Spectrometer). Electrical characterization was performed using a Keithley 2602B source meter to record current‐voltage (*I–V*) curves. The photon flux incident on the photodetector was calculated based on several geometric and radiometric parameters: the active area of the detector, the emitting area of the blackbody, the distance between the two, and the spectral distribution of the blackbody radiation. This approach ensured accurate determination of the photon flux. The spectral responsivity was then derived from the measured photocurrent and the calculated photon flux, providing comprehensive insight into the detector's response across different wavelengths. The EQE were measured by the Quantum Efficiency Measurement System (Lightsky Technology Ltd.) Atomic force microscopy images were taken in the ScanAsyst mode on Oxford Cypher 5. Transmission electron microscopy (TEM) images of NCs were obtained using a JEOL JEM‐2100F microscope. Scanning electron microscopy (SEM) measurements of pristine and patterned films were carried out on a Hitachi SU‐08010 microscope at 10 kV.

## Author Contributions

K.W. and X.T. developed the concepts. K.W. and W.L. performed device design, fabrication, and characterization. C.B., W.L., M.H., and X.Z. fabricated the single‐element device and FPA imagers. Y.L., C.B., Y.T., and C.L. collected data from the FPA imager and performed the data analysis. S.T., Y.L., Q.H., and G.M. contributed to the device fabrication. K.W. and X.T. provided tools and supervised the research. X.T. and K.W. co‐wrote the manuscript. All authors have given approval to the final version of the manuscript.

## Conflicts of Interest

X.T. and Y.L. serve as co‐founders and shareholders at XinIR Technology (Beijing) Co., L., Beijing 101102, China. The other authors declare no competing interests.

## Supporting information




**Supporting File**: advs73906‐sup‐0001‐SuppMat.docx.

## Data Availability

The data that support the findings of this study are available from the corresponding author upon reasonable request.
