# Supplementary material for: High‐Resolution Multispectral Photovoltaic Imagers from Visible to Short‐Wave Infrared
Source: Adv Sci (Weinh). 2026 Jan 20;13(18):e19991. doi: 10.1002/advs.202519991 (PMC13042821; doi:10.1002/advs.202519991)
Supplement: Supplementary file 1 — Supporting File: advs73906‐sup‐0001‐SuppMat.docx. [file ADVS-13-e19991-s001.docx]

**Supporting information**

High-Resolution Multispectral Photovoltaic Imagers from Visible to Short-Wave Infrared

*Wanqing Li, Cheng Bi, Min He, Xiaolong Zheng, Yuning Luo, Yimei Tan, Chenxi Liu, Salihuojia Talanti, Yanfei Liu, Ge Mu, Qun Hao, Kangkang Weng and Xin Tang*

**Section S1**. **Basic characteristics of organic BHJs and CQDs.**

**Synthesis of PDNBO polymer**

N,N′-Bis(2-ethylhexyl)-1,7-dibromo-3,4,9,10-perylene diimide (772mg, 1 mmol) and 1,1′-(1,6-Hexanediyldi-5,2-thiophenediyl)bis[1,1,1-trimethylstannane] (576mg, 1mmol) were added into a 100 mL Schlenk tube. Upon stirring, the monomers were dissolved in 50 mL of anhydrous toluene and degassed with nitrogen for 15min. Pd_2_(dba)_3_ (10mg) and tris(o-tolyl) phosphine (15mg) were added under nitrogen. The reaction was heated to 110°C and run for 10 hours. After the polymerization was complete, the mixture was taken up and precipitated into methanol. The solids were collected by a high-quality glass thimble, which was purified by Soxhlet extraction with acetone, hexane, and chloroform. The chloroform fraction was precipitated into methanol. The collected polymer was dried at 60 ℃ under vacuum(650mg,73%). Mn：14.6K; PDI:3.1.

**Synthesis of HgTe CQD**

To synthesize short-wave infrared (SWIR) HgTe CQDs with a cut-off absorption of 2.4 μm, HgCl_2_ (0.4 mmol) was first dissolved in 16 mL of oleylamine (OAM, Sigma-Aldrich, ≥97%) in a 30 mL glass vial, and the mixture was stirred at 100°C for 1 hour within a nitrogen-filled glove box to ensure complete dissolution and degassing. Following this, the temperature was adjusted to the reaction temperature and stabilized for 30 minutes. Subsequently, a solution of tellurium powder (Te, Sigma-Aldrich, 99.999%) in trioctylphosphine (TOP, Sigma-Aldrich, 97.000%, 0.4 mL) was rapidly injected into the vial, causing the clear solution to immediately turn black. The reaction was carried out at 80°C for 4 minutes to achieve the desired SWIR HgTe CQD synthesis. To quench the reaction, 3.6 mL of dodecanethiol (DDT, Sigma-Aldrich, 98%) and 1.2 mL of TOP were added to 16 mL of tetrachloroethylene (TCE, Aladdin, 98%). After quenching, the vial was quickly removed from the glove box and cooled to room temperature. The solution was then precipitated by adding an equal volume of isopropanol (IPA), followed by centrifugation at 6000 rpm for 5 minutes. Finally, the precipitate was resuspended in 2 mL of chlorobenzene and stored at room temperature for further use. As shown in the transmission electron microscope (TEM) images (Fig. S2), the HgTe CQDs with a cut-off absorption of 2.4 μm exhibit a mean size of 4.2 nm.


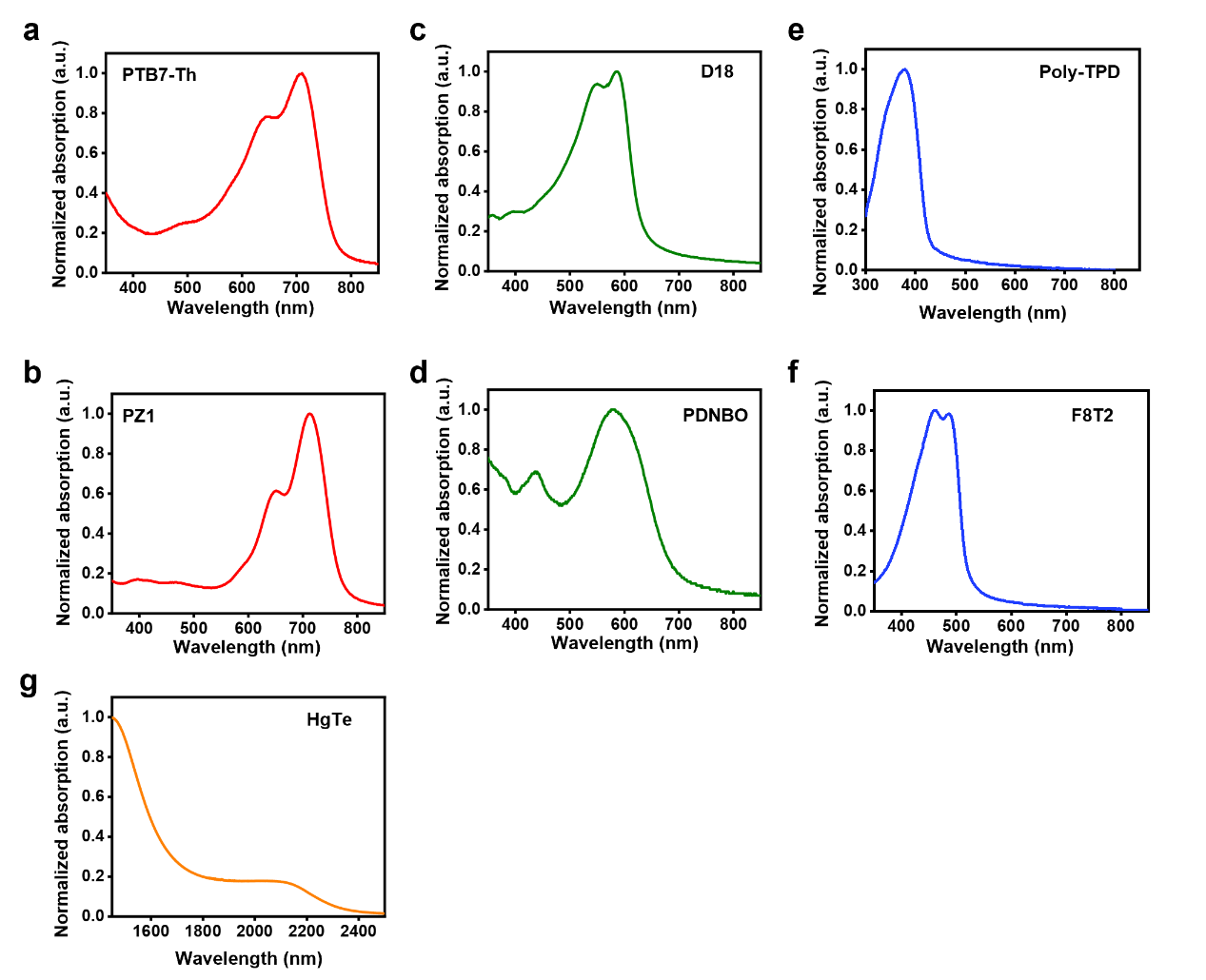


**Figure S1.** Absorption of materials used for four spectrum imaging detectors.

**Figure S2.** The low-magnification (a) and high-resolution (b) TEM images of HgTe CQDs. **c,** The size distribution of the HgTe CQDs.

**Section S2**. **Direct photolithography of BHJs and CQD devices.**


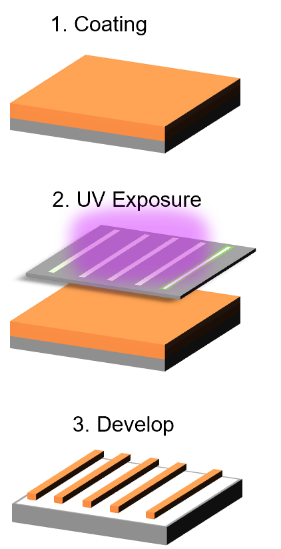


**Figure S3.** The schematic illustration of direct photopatterning process.


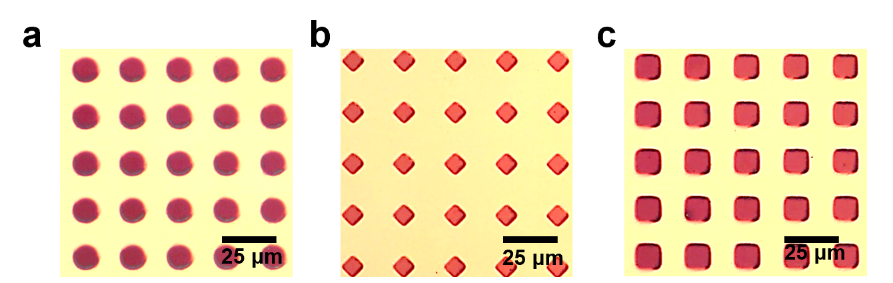


**Figure S4.** Images of different blue BHJ (Poly-TPD:F8T2) patterns including (a) circular array (b) diamond array and (c) square array.


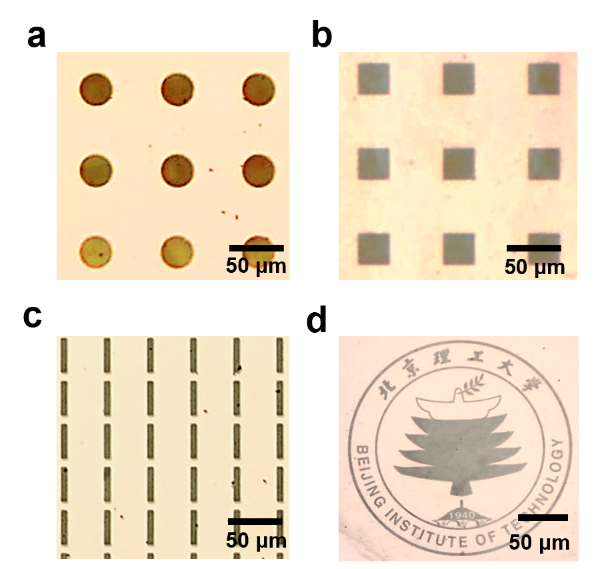


**Figure S5.** Images of different green BHJ (D18:PDNBO) patterns including (a) circular array (b) square array (c) line array and (d) complex logo.


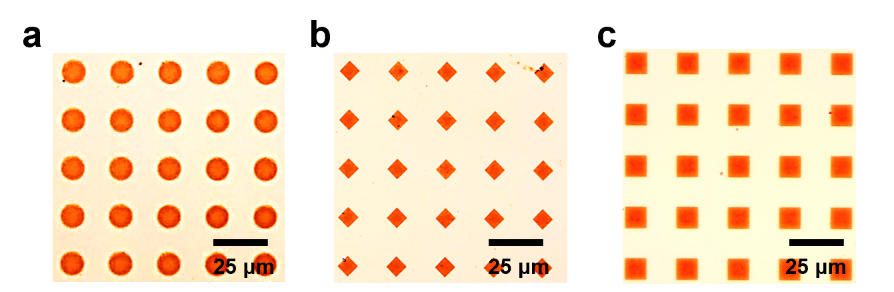


**Figure S6.** Images of different red BHJ (PTB7-Th: PZ1) patterns including (a) circular array (b) diamond array and (c) square array.


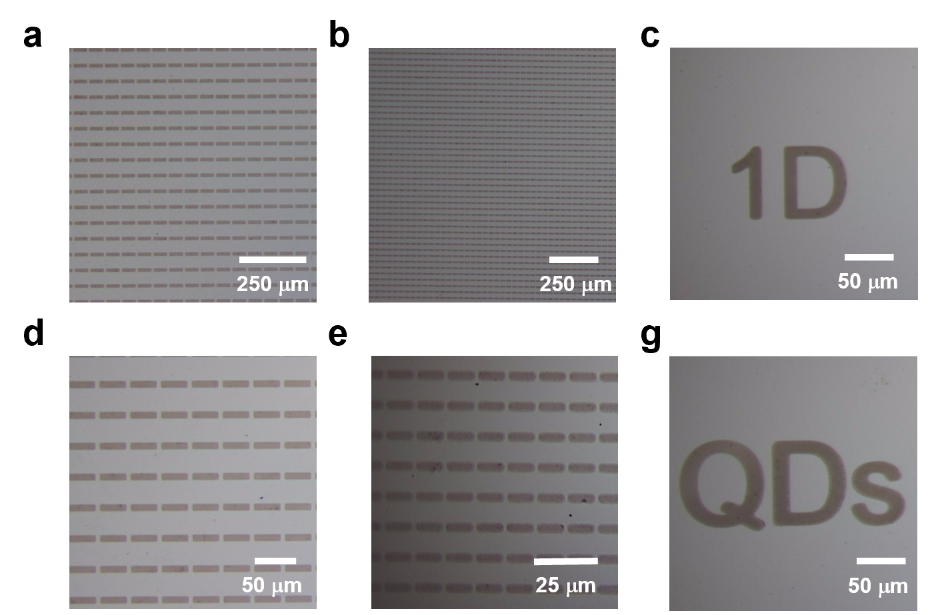


**Figure S7.** Images of different HgTe CQD patterns including (a,b,d,e) different line array and (c,g) alphabet patterns.


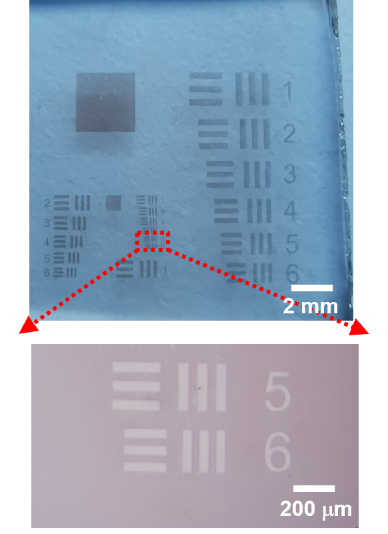


**Figure S8.** Photograph of patterned HgTe CQDs on glass. The pattern is 1951 US Air Force Target.


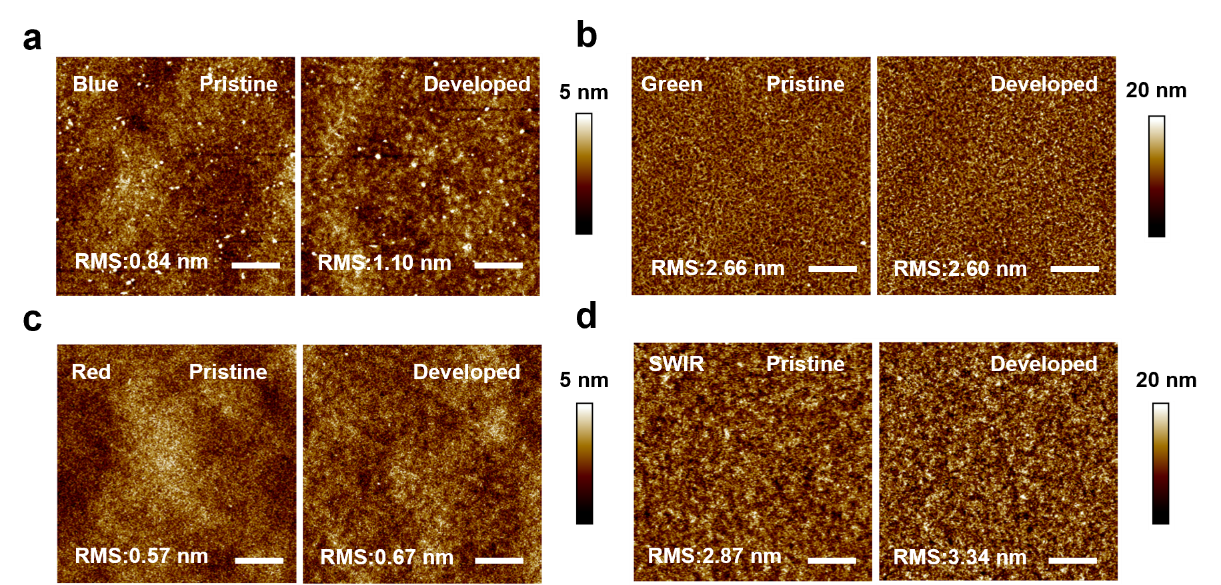


**Figure S9.** AFM images of pristine and patterned films of four-color materials. **a,** Blue. **b,** Green. **c,** Red. and **d,** SWIR. The scale bars: 2 μm.


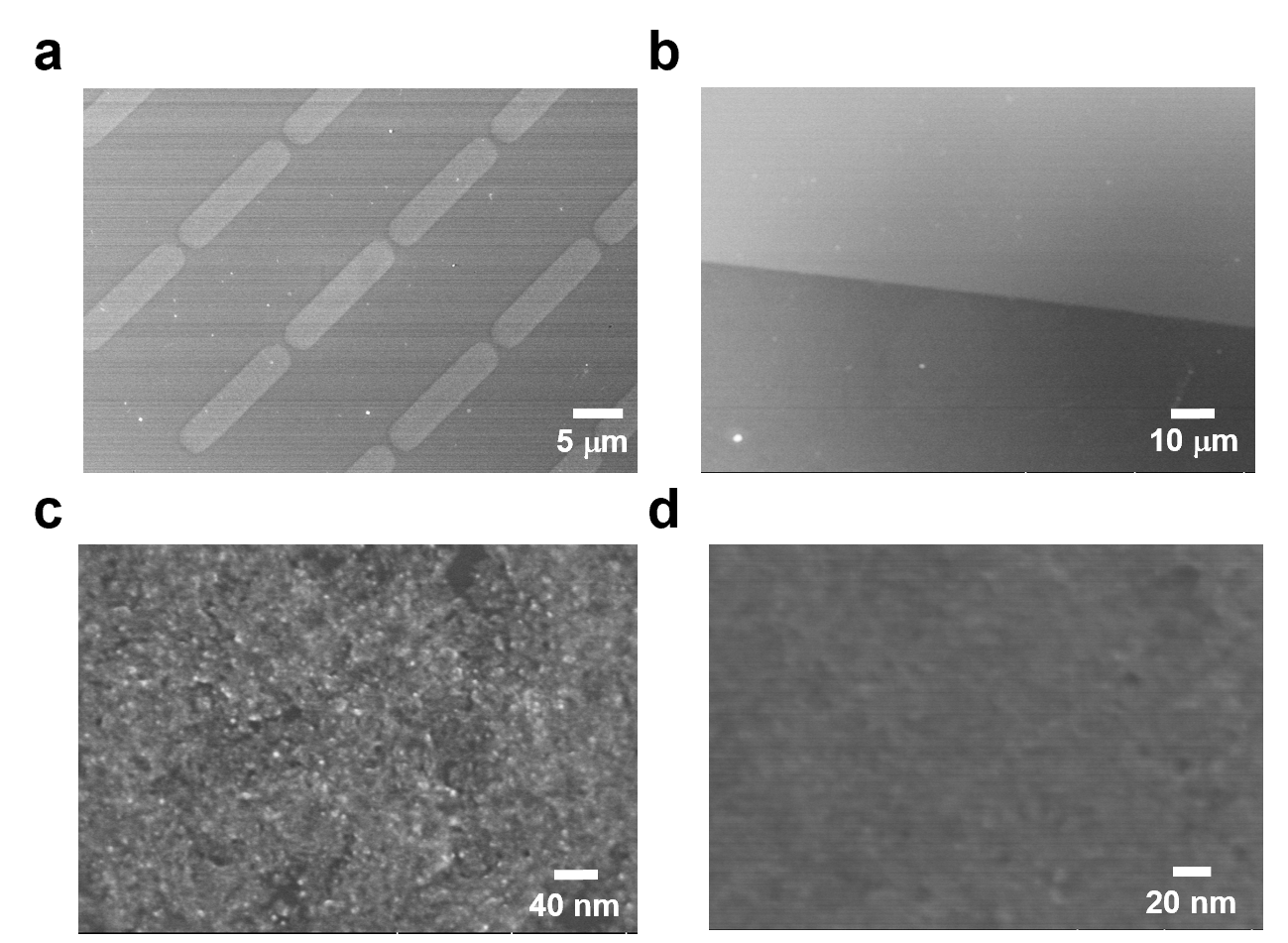


**Figure S10. a,** SEM image of a HgTe CQD line patterns. **b,** SEM image of a HgTe CQD line patterns magnified at the edge. **c, d,** Top-view SEM images of photopatterned HgTe CQD films.

**Section S3**. **The performance of R, G, B and SWIR single-element devices.**


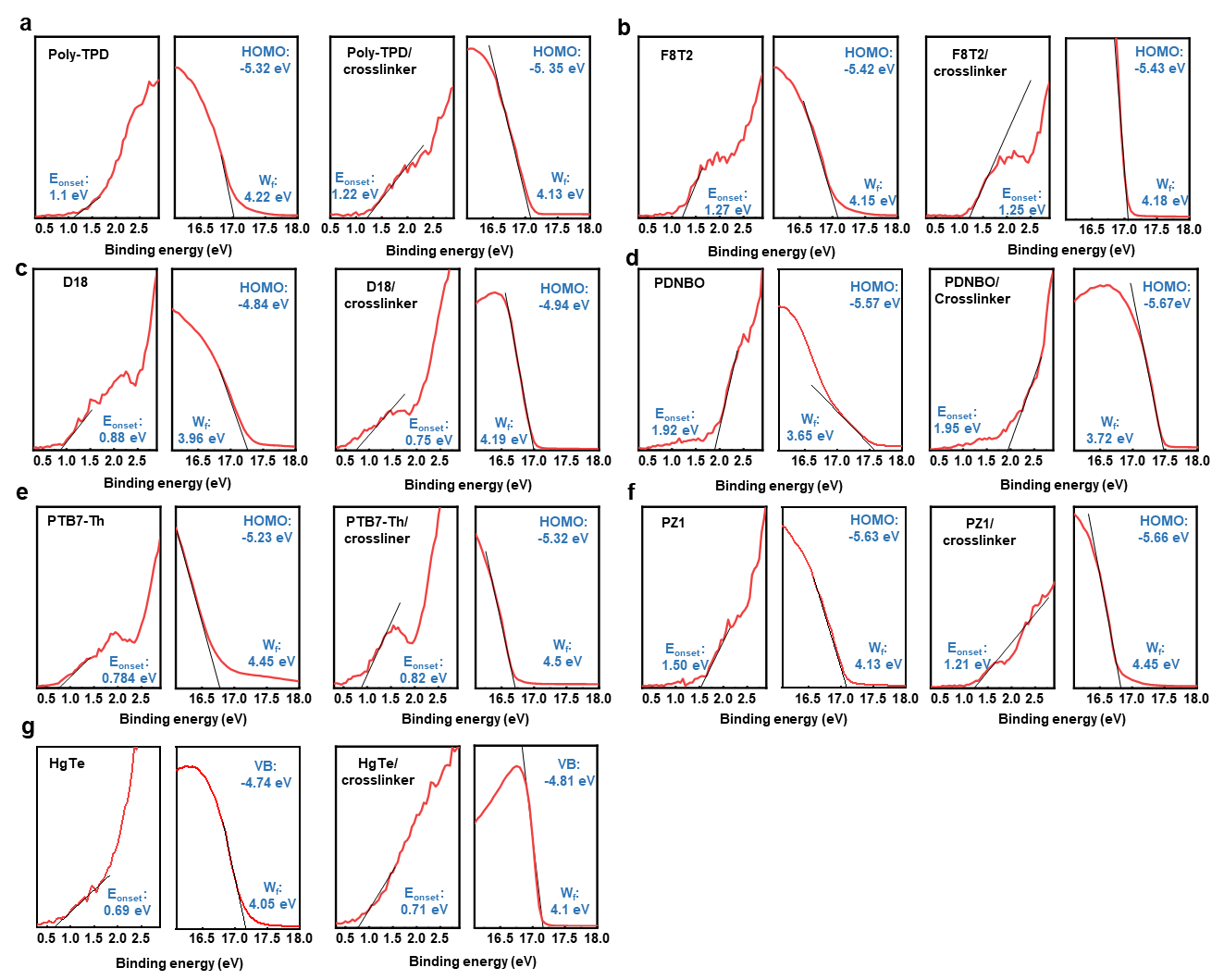


**Figure S11. UPS spectra of materials used in the detector.** The Fermi energy level (*E*_F_) was obtained directly from the UPS data and calculated by the following equation, *W*_F_ = ℎʋ − 𝐸_cutoff_. The HOMO energy calculated by the following equation, E(𝐻𝑂𝑀𝑂) = ℎʋ−(𝐸_𝑐𝑢𝑡𝑜𝑓𝑓_ −𝐸_𝑜𝑛𝑠𝑒𝑡_).

**Table S1.** The energy level offset of organic BHJ components and HgTe CQDs.

|  | **Materials** | **HOMO/VB (eV)** | **LUMO/CB (eV)** | **Bandgap (eV)** |
| --- | --- | --- | --- | --- |
| **Blue** | Poly-TPD | -5.35 | -2.45 | 2.9 |
|  | F8T2 | -5.43 | -3.06 | 2.37 |
| **Green** | D18 | -4.96 | -2.98 | 1.98 |
|  | PDNBO | -5.67 | -3.86 | 1.81 |
| **Red** | PTB7-Th | -5.32 | -3.71 | 1.61 |
|  | PZ1 | -5.66 | -4.06 | 1.60 |
| **Crosslinker** | / | -5.8 | -2.54 | 3.35 |
| **SWIR** | HgTe | -4.81 | -4.35 | 0.46 |


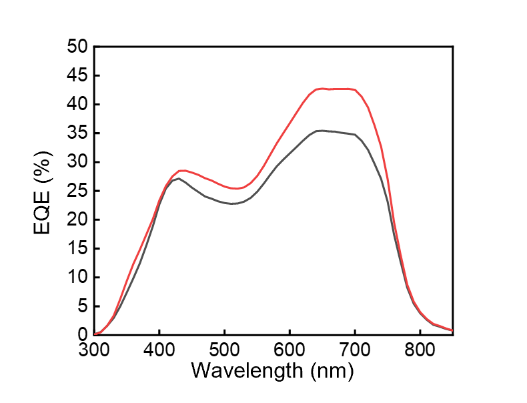


**Figure S12.** EQE spectra of pristine and developed red-detector.


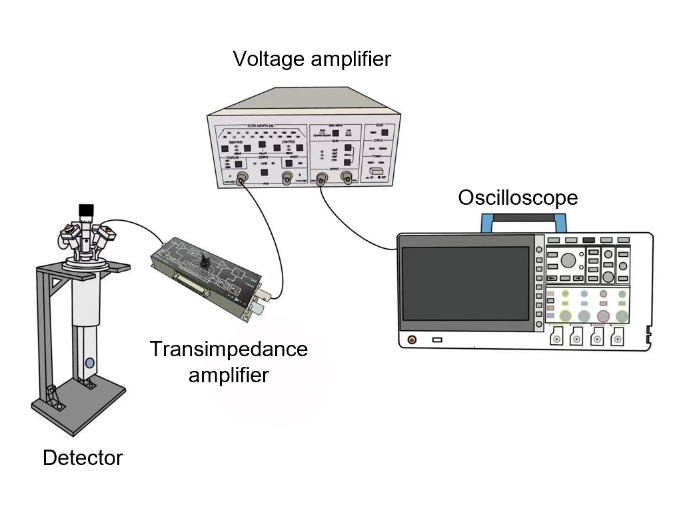


**Figure S13.** Schematic diagram of the RMS noise measurement process.

The relationship of spectral noise density (*I_n_*) and RMS noise (*I_RMS_*) is shown as follows:

$$\begin{aligned} \text{I}_{\text{RMS}}\text{=}\sqrt{\int_{\text{low}}^{\text{high}} {\text{I}_{\text{n}}\left( \text{f} \right)}^{\text{2}}\text{df}}\#\text{(S1)} \end{aligned}$$

where *I_n_* (*f*) is the spectral noise density at frequency *f*, *f_low,_* and *f_high_* are the lower and upper limits of the frequency range over which the noise is measured, respectively. The RMS noise is measured with a low-pass filter with a cut-off frequency at 1KHz, as shown in **Figure S13**. According to **Equation S1**, the spectral noise density could be calculated.

**Figure S14a** compares *J-V* characteristics of pristine and patterned blue photodetectors under 455 nm illumination (1 mW cm⁻²) and dark conditions. The patterned blue PDs exhibit a dark current density of 1.36×10⁻⁸ A cm⁻² at 0 V bias, comparable to that of pristine devices (7.4×10⁻⁹ A cm⁻²). Zero-bias EQE measurements (**Fig. S14b**) reveal a peak value of 3.1% for patterned PDs, matching the performance of pristine devices (3.5%). Both device configurations show nearly identical peak responsivity values of 0.012 A W⁻¹ at zero bias. Noise characterization indicates RMS noise currents ranging from 0.07 pA to 0.13 pA for pristine and patterned devices, respectively. The specific detectivity spectra under zero bias (**Fig. S14d**) exhibit peak values of 4.2×10^10^ Jones and 2×10^10^ Jones for pristine and patterned PDs, demonstrating comparable detection capabilities despite structural modifications. Similarly, the pristine and patterned green photodetectors demonstrate comparable performance metrics.


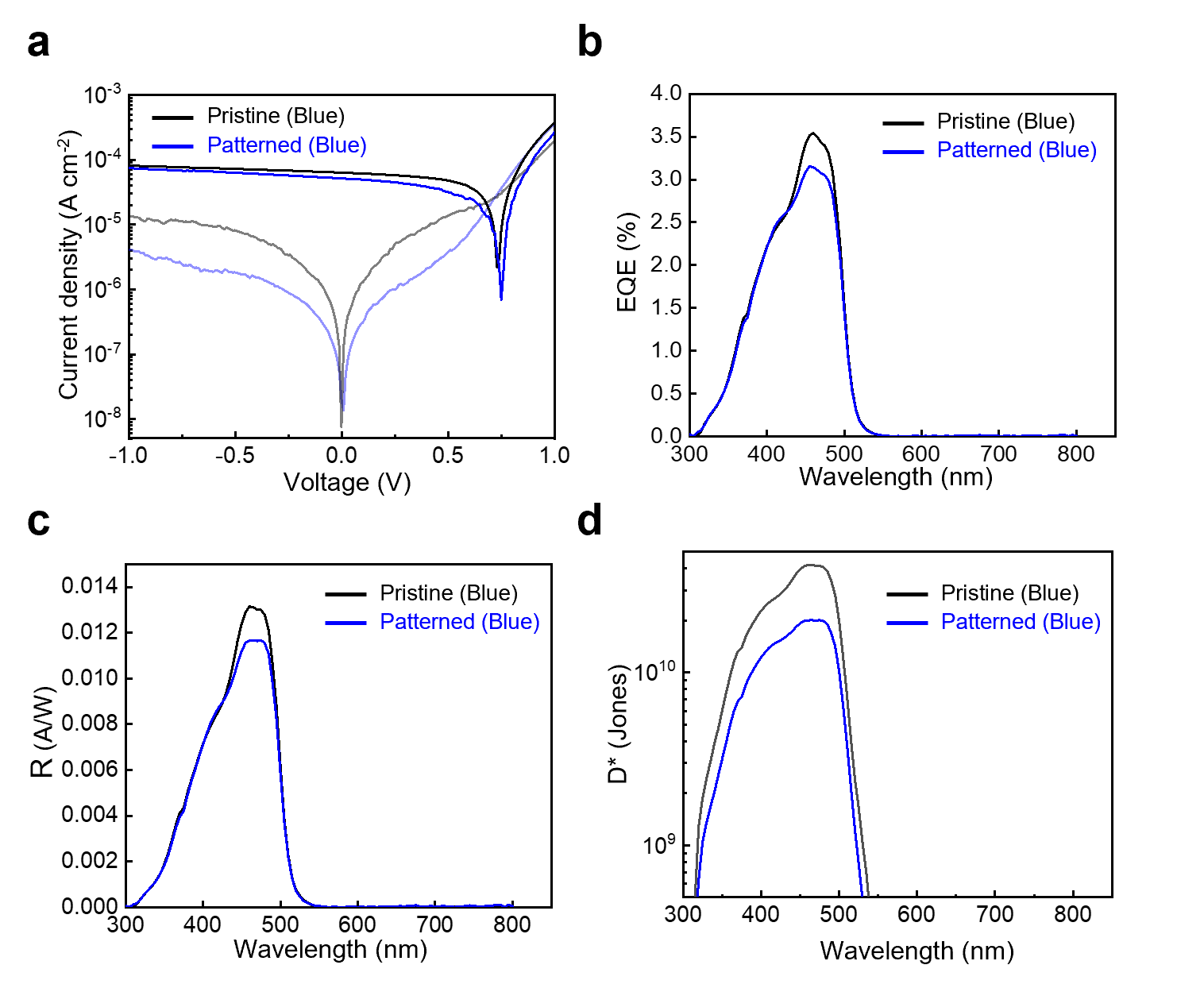


**Figure S14. Blue-detectors performance. a,** Current density–voltage (*J* – *V*) curves of pristine and patterned detector in dark and under illumination. Dark current density: semi-transparent curves. Photocurrent density: solid curves. **b,** EQE spectra of pristine and developed detector. **c,** Spectral responsivity of pristine and patterned detectors. **d,** Detectivity spectrum of pristine and patterned detectors.

As illustrated in **Figure S15**, the patterned green PDs exhibit a dark current density of 1.36×10⁻⁸ A cm⁻² at 0 V bias, comparable to that of pristine devices (7.4×10⁻⁹ A cm⁻²). Zero-bias EQE measurements (**Fig. S15b**) reveal a peak value of 22% for patterned PDs, matching the performance of pristine devices (22%). Both device configurations show nearly identical peak responsivity values of 0.1 A W⁻¹ at zero bias. Noise characterization indicates RMS noise currents ranging from 0.09 pA to 0.28 pA for pristine and patterned devices, respectively. The specific detectivity spectra under zero bias (**Fig. S15d**) exhibit peak values of 1.1×10^11^ Jones and 4.4×10^10^ Jones for pristine and patterned PDs.


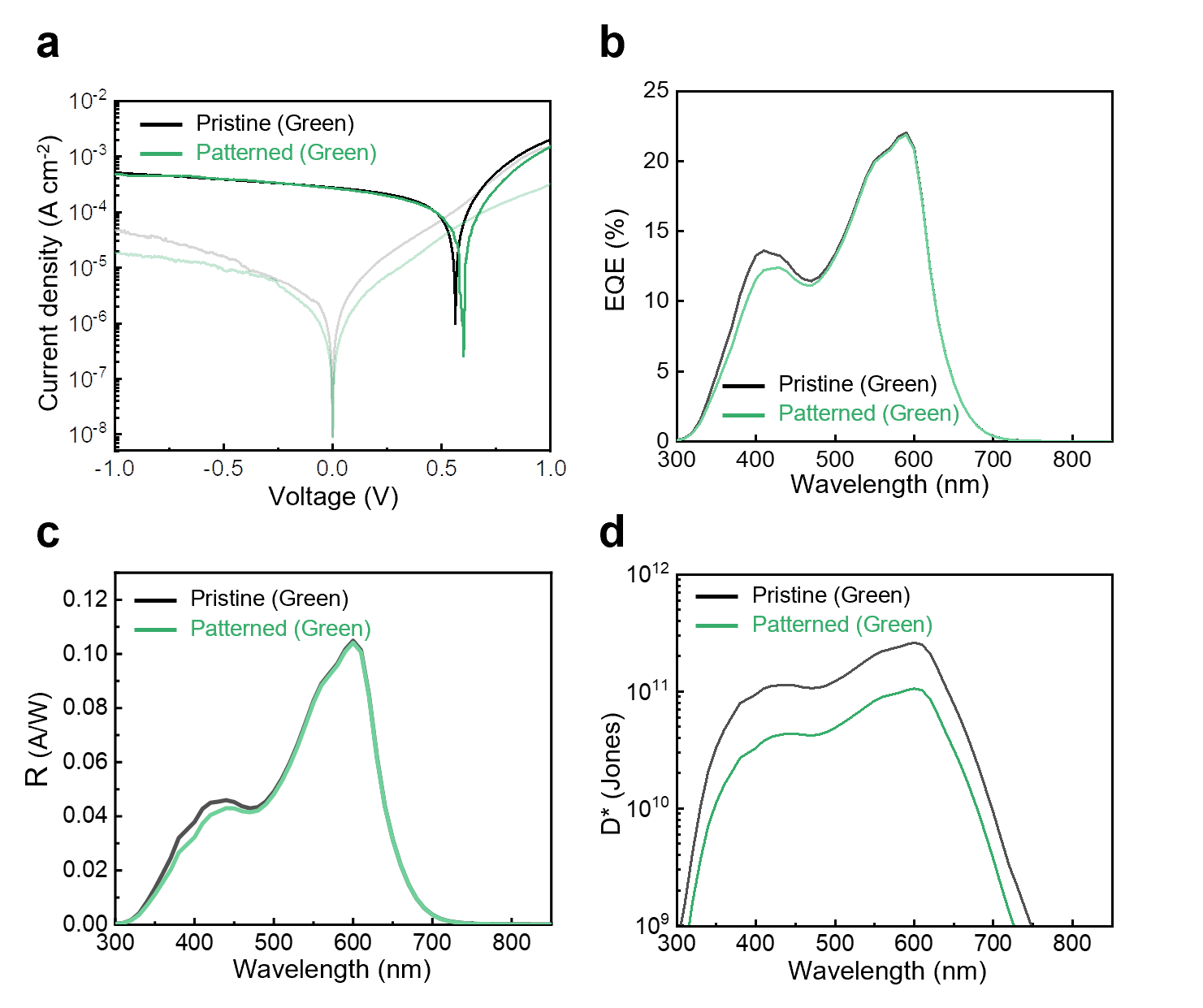


**Figure S15. Green-detectors performance. a,** Current density–voltage (*J* – *V*) curves of pristine and patterned detector in dark and under illumination. Dark current density: semi-transparent curves. Photocurrent density: solid curves. **b,** EQE spectra of pristine and developed detector. **c,** Spectral responsivity of pristine and patterned detectors. **d,** Detectivity spectrum of pristine and patterned detectors.


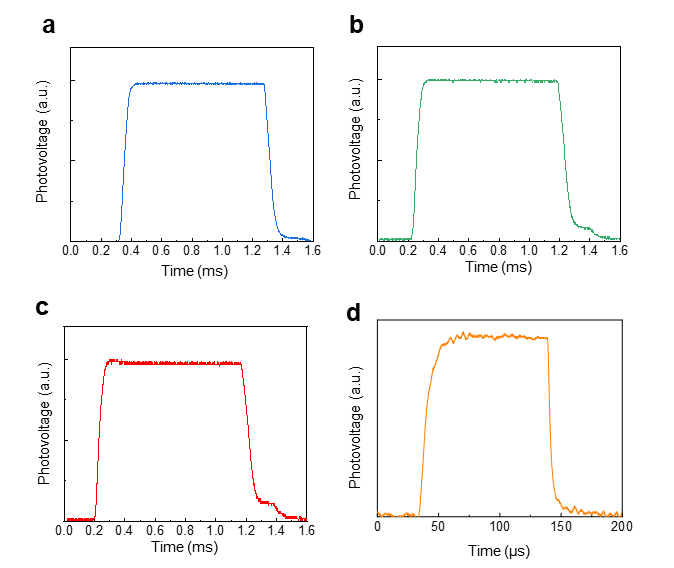


**Figure S16.** Response time of patterned photodetectors. **a,** Blue photodetectors. **b,** Green photodetectors. **c,** Red photodetectors. **d,** SWIR photodetector.

**Figure S17. SWIR-detectors performance. a,** Schematic of the SWIR detector performance measurement process. **b**, Schematic of device structure of SWIR detector. Cross-sectional TEM image of the pristine (**c**) and patterned (**d**) SWIR detector. The scale bar is 100 nm. Current density–voltage (*J* – *V*) curves (**e**), EQE spectrum (**f**), responsivity spectrum (**g**) and detectivity spectrum (**h**) of pristine and patterned detector.

**Figure S18. FTIR spectroscopic analysis of surface chemistry evolution during ligand exchange.** The spectra compare the as-synthesized quantum dots capped with DDT and those after ligand exchange with EDT. The most prominent change is the strong attenuation of the characteristic C–H stretching modes (between 2800-3000 cm⁻¹), which directly indicates a substantial reduction in surface aliphatic carbon chains due to the replacement of DDT. This successful exchange to a compact EDT layer is critical for reducing inter-dot distance and improving charge transport in CQD solids.

**Figure S19.** The noise spectral density at 0 V of red-detector (a), green-detector (b), blue-detector (c) and SWIR-detector (d).

**Table S2.** The RMS noise currents and *D** of four types of detectors.

| Detector | | I_RMS_(1-1000Hz) (pA) | D^*^_max_ (Jones) |
| --- | --- | --- | --- |
| Red | Pristine | 0.02 | 1.64×10^12^ |
|  | Crosslinked | 0.04 | 1.78×10^12^ |
| Green | Pristine | 0.12 | 1.96×10^11^ |
|  | Crosslinked | 0.31 | 9.53×10^10^ |
| Blue | Pristine | 0.25 | 3.27×10^10^ |
|  | Crosslinked | 4.21 | 1.04×10^10^ |
| SWIR | Pristine | 1.3 | 1.46×10^11^ |
|  | Crosslinked | 2.1 | 8×10^10^ |

**Section S4**. **RGB-SWIR quad-spectral imagers performance characterization.**


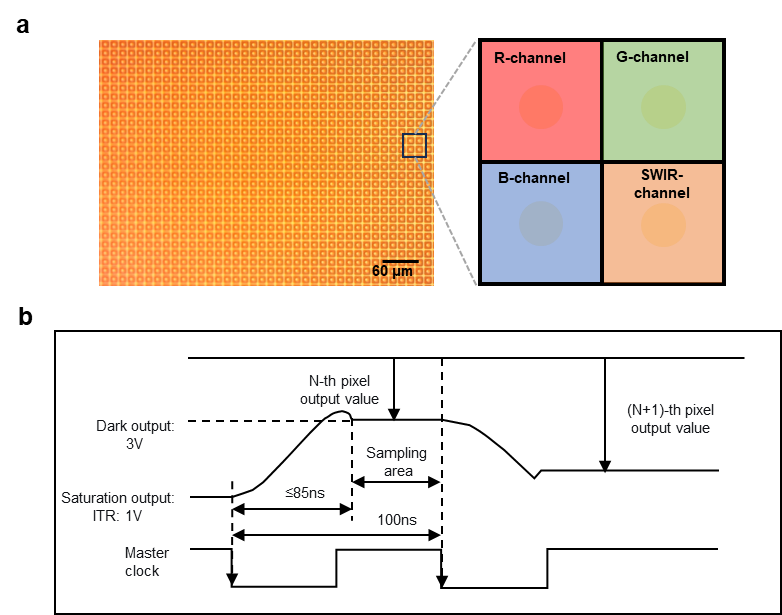
The silicon readout circuits (ROICs) with 640 × 512 pixels and 15 μm pixel pitch were used as the substrate for FPA imagers (**Figure S20a**). A bias voltage is applied between the pixel electrode and the ground electrode. The layout of ROICs is presented in **Figure S22**. The function and typical setting of pins are shown in **Table S3**. Analog output characteristics is illustrated in **Figure S20b**. The global shutter (Snapshot) operation is crucial for capturing dynamic scenes without motion artifacts. The availability of ITR modes offers a valuable trade-off between full-well capacity (5.3 Me-) and output swing (2V), allowing optimization for high dynamic range or specific frame rate. The four-channel output structure with a single-tap readout rate ≥10 MHz enables high frame rates while distributing the bandwidth, which is beneficial for maintaining signal integrity and low noise. The readout circuit outputs include four analog detector outputs (OUT1/2/3/4) and two digital outputs (DATAVALID and ERROR). The analog outputs can drive a maximum equivalent load presented by a parallel connection of a resistor no less than 100 kΩ and a capacitor no greater than 15 pF. The pixels are read out at the master clock frequency, and the analog output voltage range is related to the supply voltage, VAB, and other bias conditions.

**Figure S20.** a, The geometric arrangement of pixels in a quad-channel imager. b, Analog Output Characteristics (Load: R ≥ 100 kΩ, C ≤ 15 pF).

The 4-channel spectral chip achieves pixel-level spectral sensing through direct photopatterning method that directly forms cyclic 2×2 spectral channel sensing windows in the pixel array. Each individual pixel position within these windows is integrated with distinct spectral-sensitive materials responsive to specific wavelength bands, analogous to microlens arrays in conventional visible-light CMOS sensors but fabricated through simplified photolithographic patterning of photosensitive materials rather than complex MEMS-based microlens manufacturing. This design enables simultaneous acquisition of four spectral channels within a single snapshot, forming hyperspectral imaging capabilities. However, the planar architecture inevitably compromises spatial resolution, necessitating the implementation of a coordinate attention-enhanced super-resolution model to reconstruct high-resolution spectral images while mitigating spatial-domain information loss. The proposed model incorporates positional awareness through attention mechanisms to capture textural details, utilizing hybrid operations including global average pooling and global maximum pooling to extract comprehensive contextual features. These operations enhance inter-pixel correlations and strengthen feature representation across both spatial and channel domains.

The evolution of SR architectures, from the foundational SRCNN establishing convolutional mapping between low/high-resolution images, to subsequent innovations like ESPCN, EDSR, SRGAN, and SwinIR incorporating attention mechanisms and generative adversarial networks, has progressively improved texture realism in reconstructed images. For our multi-spectral chip architecture, the customized SR model implements three-stage processing: shallow feature extraction, deep feature extraction, and image reconstruction. Addressing the spatial coupling of four spectral channels and considering the enlarged pixel dimensions of infrared readout integrated circuits (ROIC, 15μm pixel pitch) compared to visible-light CMOS, the model processes multi-channel spectral inputs collectively rather than individually.

In this method, spectral information from different spectral channels and spatial pixel features is combined, tailored to the detector structure. In the deep feature extraction layer, the Convolutional Block Attention Module (CBAM) is adopted^1,2^, resulting in the formation of a fused channel-spatial spectral feature super-resolution model (FCSFSR). In the process of extracting channel spectral features, global spectral information in the spatial domain is captured using global maximum pooling and global average pooling. The input spectral features $F$ are transformed into $F_{max}^{c}$ and$F_{avg}^{c}$, and then passed through a Conv-ReLU-Conv (MLP) block to obtain $Avg_{c}$and $Max_{c}$, respectively. These two values are then summed and passed through a Sigmoid function to obtain the feature weights. The input features are then multiplied by the weights to obtain the channel spectral feature $F_{c}$.

For spatial spectral features, the aim is to emphasize the feature relationships between different spectral channels and enhance the information of related spectral channels. In the spatial feature extraction process, global maximum pooling and global average pooling are also used to extract global information, but the calculation direction differs from that in the channel feature extraction. The operation is performed along the channel dimension, obtaining the average feature $F_{max}^{s}$ and the maximum feature $F_{avg}^{s}$ for different channels. These two features are then concatenated to form a dual-layer feature, which is subsequently mapped to a one-dimensional feature $F_{avg\&max}$ through Conv-ReLU-Conv (MLP). The Sigmoid function is then used to generate spatial feature weights, and finally, the input features are multiplied by the weights to obtain the spatial spectral feature $F_{s}$. The channel spectral feature $F_{c}$ and the spatial spectral feature $F_{s}$ are added together to obtain the fused channel-spatial spectral feature. The structure of FCSFSR is shown in **Figure 4d**.

During the network training process, input data is acquired using a four-channel spectral detector. Prior to feeding the data into the network, preprocessing steps such as channel separation are performed. The original high-resolution images serve as target inputs, while low-resolution input images are generated by directly downsampling the originals. This downsampling method aligns with the actual process of resolution reduction. To enhance the model's generalization capability, data augmentation techniques (e.g. rotation and scaling) are employed during training to increase the diversity of the training dataset, thereby effectively preventing overfitting. Regarding the choice of loss function, the L2 loss function (mean squared error) is utilized. This loss function aids in optimizing the peak signal-to-noise ratio (PSNR), resulting in smoother images and is suitable for structural recovery.

In this model, Spectral inputs initially pass through channel attention to derive channel-wise weighting coefficients, adaptively emphasizing critical spectral bands. Concurrently, spatial attention generates pixel-level importance maps highlighting texturally significant regions. These dual attention features are concatenated and processed through an MLP-based fusion network that globally models channel-wise and spatial descriptors. The integrated features undergo element-wise summation before final upsampling through learned transposed convolutions to generate high-resolution hyperspectral outputs.

This architecture achieves synergistic enhancement of spectral fidelity and spatial resolution by maintaining inter-channel correlations while adaptively weighting both spectral and spatial information domains. The attention-driven feature fusion mechanism effectively compensates for resolution limitations inherent in the planar multi-spectral sensor architecture.


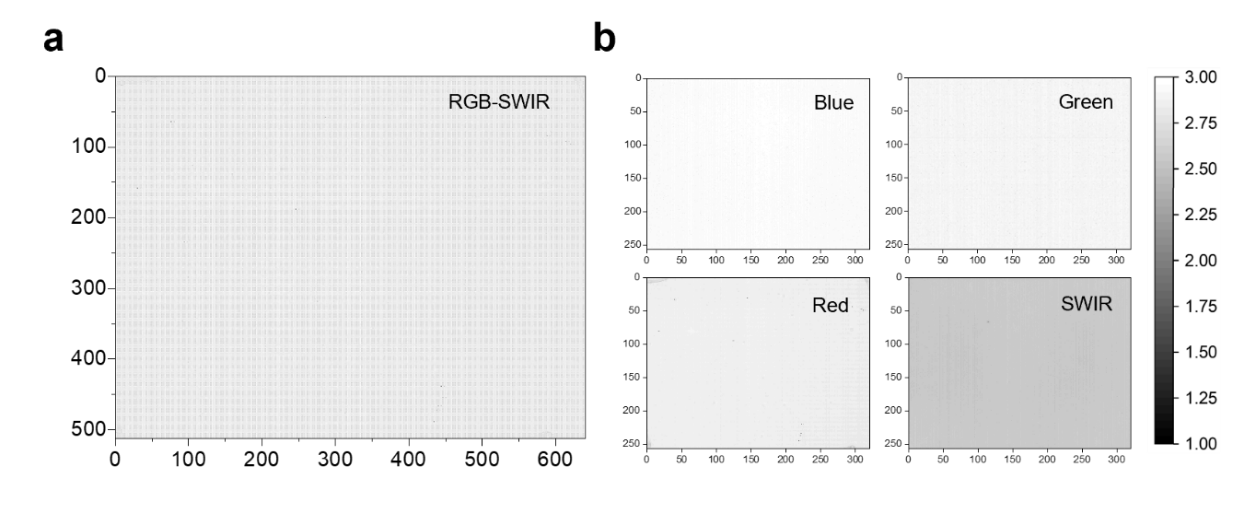


**Figure S21. a,** The photocurrent mapping of the quad-spectral FPA imager. **b,** Channel-specific photocurrent mapping images (256×320 resolution) obtained through spectral decomposition for individual red, green, blue, and SWIR detection channels.


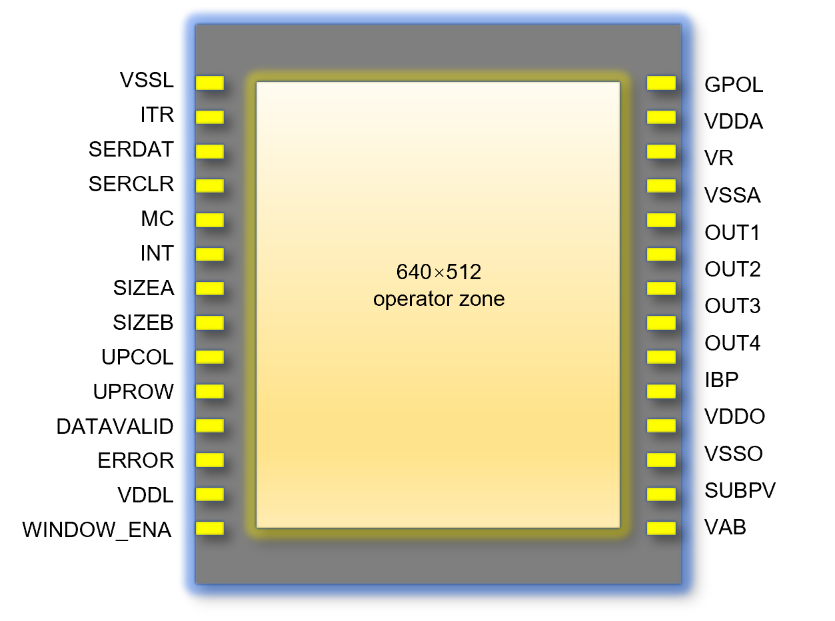


**Figure S22.** The layout of ROICs.

**Table S3.** The function and typical setting of pins.

| Name | I/O | Define | Type |
| --- | --- | --- | --- |
| GPOL | I | Bias control | Analog |
| VDDA | I | Analog Power | Power |
| VSSA | I | Analog Ground | Ground |
| VDDO | I | Output Power | Power |
| VSSO | I | Output Ground | Ground |
| VR | I | Reset Power | Analog |
| VDETCOM | I | Common terminal bias | Power |
| IBP | O | Detector bias adjustment | Analog |
| OUT1/2/3/4 | O | Output | Analog |
| DATAVALID | O | Readout synchronization | Digital |
| ERROR | O | Error signal | Digital |
| MC | I | Clock | Digital |
| INT | I | Integration time | Digital |
| SIZEA | I | Window control | Digital |
| SIZEB | I | Window control | Digital |
| UPCOL | I | Image rotation | Digital |
| UPROW | I | Image rotation | Digital |
| ITR | I | Exposure readout mode selection | Digital |
| SERDAT | I | Window coordinate input interface | Digital |
| SERCLR | I | Serial register reset | Digital |
| WINDOW_ENA | I | Arbitrary window enablement | Digital |
| VAB | I | Overflow protection bias voltage | Analog |
| VDDL | I | Digital Power | Power |
| VSSL | I | Digital Ground | Ground |

The architectures of the FPA circuits are presented in **Figure S23**. Incident photons are absorbed by a photosensitive layer, which converts them into electrons. These electrons then move to the pixel circuits, forming a photocurrent that is directly injected into each pixel’s capacitive nodes. These nodes are designed to capture and hold the weak signals, storing the electronic charge to enhance signal sampling and retention. Subsequently, through row and column selector controllers, the voltage values of each pixel are output to the out pin following specific output rules.


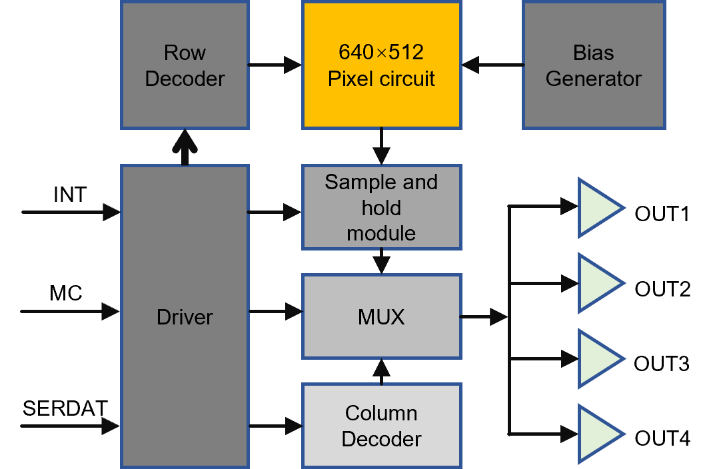


**Figure S23.** The schematic diagram of FPA circuit architectures.

Without illumination, the dark current *I_dark_* with integration time of 80ms can be calculated by^3,4^:

$$\begin{aligned} I_{dark}=\frac{f\cdot\left( V-V_{dark} \right)\cdot e}{t_{integral}}\#(\text{S2)} \end{aligned}$$

where *e* is the elementary charge. The maximum range of output voltage *V_max_* is 3V, the *V*_dark_ are 2.986, 2.981, 2.969 and 2.897 V for blue, green, red and SWIR detectors, respectively. The conversion factor *f* from voltage to number of charges is ~2.2083Me^-^/V.

For the FPA imager, the root-mean-square (RMS) noise voltage (*V_rms_*) for each pixel is derived from the acquisition of 100 frames under dark conditions.

$$\begin{aligned} \text{V}_{\text{rms}}\left( \text{i, j} \right)\text{=}\sqrt{\left\{ \frac{\text{1}}{\text{F}}\sum_{\text{f}\text{ }\text{=1}}^{\text{F}} \left\{ {\bar{\text{V}}}_{\text{dark}}\left( \text{i, j} \right)\text{ - }\text{V}_{\text{dark}}\left( \text{i, j, f} \right) \right. \right\}^{\text{2}}}\# \\ \end{aligned}\text{(S3)}$$

where $\text{i}$ and $\text{j}$ denote the row and column indices of a pixel,$\text{ }\text{V}_{\text{dark}}\left( \text{i, j, f} \right)$ is the output voltage of the pixel located at the $\text{i}^{th}$ (row) and $j^{th}$ (column) under dark conditions during the $f^{th}$ frame, and ${\bar{\text{V}}}_{\text{dark}}\left( \text{i, j} \right)$ signifies the mean output voltage of pixel $\left( \text{i, j} \right)$ averaged over $\text{F}$ captured frames with $\left( \text{F}\text{=}\text{ }\text{100} \right)$.

**Section S5:** **Quantitative Evaluation of FCSFSR**

**Experimental Results and Analysis of FCSFSR**

We provide a comprehensive quantitative evaluation of our proposed FCSFSR model. To objectively assess the reconstruction quality, we utilize three standard metrics on the test dataset: Peak Signal-to-Noise Ratio (PSNR), Structural Similarity Index (SSIM), and Naturalness Image Quality Evaluator (NIQE). As presented in **Table S4**, FCSFSR achieves a PSNR of 34.61 dB and SSIM of 0.9844, demonstrating its superior capability in recovering spectral details compared to baseline methods.

**Table S4.** Quantitative comparison on the proposed dataset

| **Method** | **Scale** | **PSNR (dB)** | **SSIM** | **NIQE** |
| --- | --- | --- | --- | --- |
| Bicubic (Baseline) | ×2 | 28.38 | 0.8635 | 5.0168 |
| This method | ×2 | 34.61 | 0.9844 | 4.1280 |

In addition to the quantitative assessment, we conduct a qualitative evaluation to intuitively demonstrate the super-resolution performance of the proposed FCSFSR. **Figure S23** illustrates the visual comparison between the Bicubic interpolation baseline and our method on the test dataset. As observed in the zoomed-in regions, the Bicubic interpolation tends to produce unsmooth results, suffering from severe jagged artifacts and staircase effects along the edges. It fails to reconstruct continuous structural lines and the intricate textures inherent in the spectral images.

In contrast, FCSFSR effectively alleviates these jagged artifacts. Our method successfully reconstructs continuous and sharp edges, suppressing the staircase effects while preserving fine details. This results in images that are visually closer to the Ground Truth. This visual superiority validates that our parallel attention mechanism successfully captures both spatial context and channel-wise dependencies, leading to more plausible and natural reconstructions.


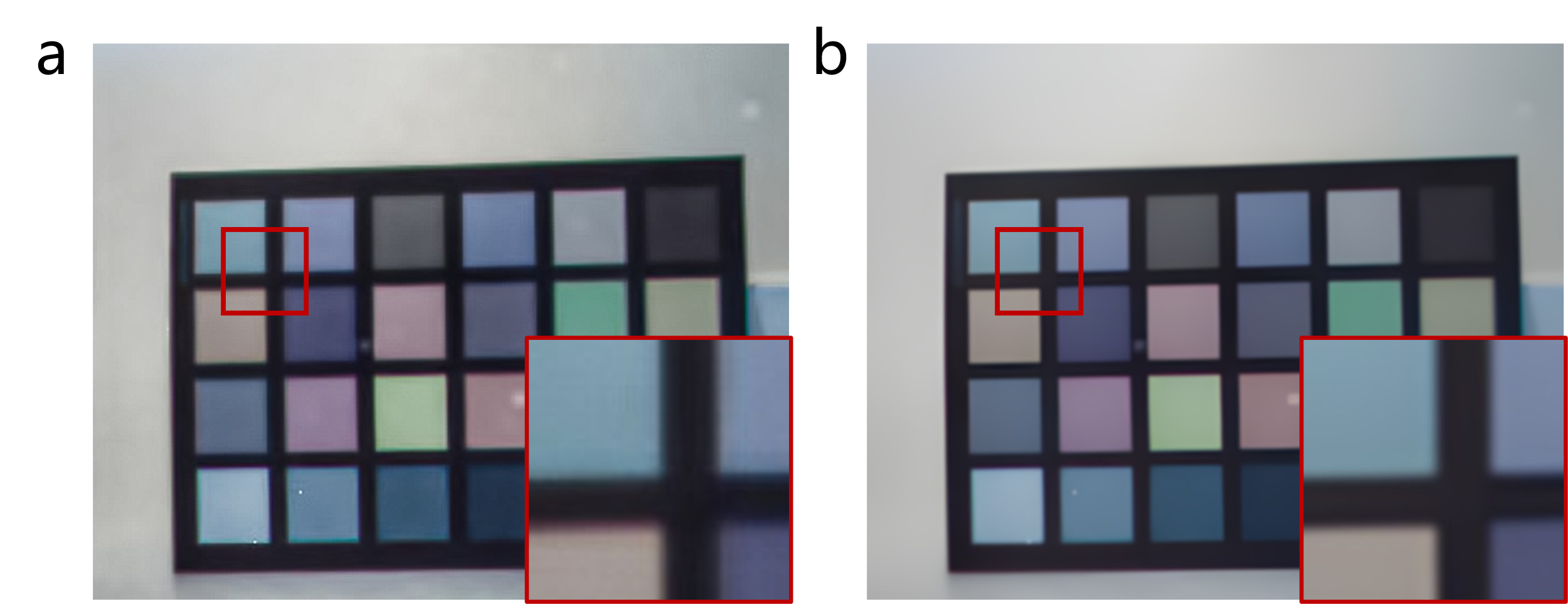
**Figure S24.** Visual comparison of super-resolution results on the test dataset. The red boxes indicate the regions of interest (ROI). (a) Bicubic Interpolation. (b) FCSFSR.

**Ablation Analysis of FCSFSR**

To investigate the effectiveness of each component in the proposed FCSFSR network, we conducted comprehensive ablation studies on the test dataset. Specifically, we analyzed the contributions of the Channel Attention Mechanism (CAM), the Spatial Attention Mechanism (SAM), and the proposed parallel fusion strategy. The quantitative results are summarized in **Table S5**.

**Table S5.** Quantitative comparison on the proposed dataset

| **Configuration** | **PSNR (dB)** | **SSIM** | **NIQE** |
| --- | --- | --- | --- |
| Baseline (No attention) | 33.41 | 0.9768 | 8.9565 |
| Baseline + CAM | 33.86 | 0.981 | 9.4399 |
| Baseline+ SAM | 34.38 | 0.9823 | 8.905 |
| Baseline + CAM + SAM (Sequential) | 34.47 | 0.9818 | 9.0869 |
| Baseline + CAM + SAM (Parallel) | 34.61 | 0.9844 | 8.5582 |

We established a baseline by excising attention modules from the standard RDB network, yielding a PSNR of 33.41 dB. Integrating CAM and SAM independently elevate performance to 33.86 dB and 34.38 dB, respectively. These consistent gains confirm that attention mechanisms are instrumental in spectral super-resolution, effectively enhancing feature selectivity while suppressing irrelevant noise. Notably, the SAM-only configuration surpasses the CAM-only variant by 0.52 dB (34.38 dB vs. 33.86 dB). This disparity underscores the predominance of spatial contextual information over inter-channel dependencies in spectral imagery, attributed to the necessity of preserving high-frequency geometric structures and textural details inherent in the data. Regarding fusion strategies, the sequential stacking (analogous to CBAM) attains a PSNR of 34.47 dB. In contrast, our proposed Parallel configuration secures the highest aggregate performance (34.61 dB PSNR, 0.9844 SSIM). The parallel architecture facilitates the joint optimization of channel and spatial features, mitigating information loss associated with serial suppression. Furthermore, the superior NIQE score (8.5582) corroborates that parallel fusion not only enhances pixel-level fidelity but also yields perceptually superior reconstructions with natural textures.

**Table S6.** A summary of the optoelectronic and device properties of B-, G- R- and SWIR sensitive photodetectors.

| Photodetector | Materials | Range  (nm) | Operate voltage(V) | *I_noise_*  (pA Hz^−1/2^) | EQE_max_  (%) | R  (A/W) | D* peak (Jones) | T  (μs) | Reference |
| --- | --- | --- | --- | --- | --- | --- | --- | --- | --- |
| B | MAPb(Br _0.65_Cl_0.35_ )_3_ | 320-510 | 0 | 0.04**^a^** | 84.9 | 0.213 | 5.57×10 ^12^ | 585/531 | **5** |
|  | Cs_2_AgBiBr_6_/ SnO_2_ | 300-520 | 0 | 15.2**^a^** | 40 | 0.11 (350 nm) | 2.1×10^10^ | 2000 | **6** |
|  | C_60_:Rubrene | 300-550 | 0 | 0.0002**^a^** | 40 | 0.116(455nm) | 6.35×10^14^ | 0.861 /1.38 | **7** |
|  | F8T2:4Bx | 300-500 | -5 | 0.14**^b^** | ~10 | / | 3×10^10^ | / | **8** |
| G | PVK:SubPc | 300-620 | 0 | 0.06**^a^** | 31.91. | 0.14 | 2.40 × 10^12^ | 30000/32000 | **9** |
|  | DM-2,9-DMQA:SubPc | 450-630 | -3 | 0.13**^a^** | 57 | 0.264 | 2.03 × 10^12^ | 4.34 /4.42 | **10** |
|  | CdSe (5 nm) | 300-550 | 15 | / | / | > 10 A/W | 2.1 × 10^11^ | 270/90000 | **11** |
|  | P3HT:4Bx | 300-640 | -5 | 0.2@10kHz | ~8000 | / | 4.2× 10^12^ | / | **8** |
| R | PTB7-Th:4Bx | 300-750 | -5 | 0.18**^b^** | 200 | / | 2×10^11^ | / | **8** |
|  | PTB7:PC_71_BM:Rubren | 300-750 | 0 | ~1000 | ~80 | 0.45 | 2.53×10^11^ | 69000/82000 | **12** |
|  | CH_3_NH_3_PbI_3−x_Cl_x_ | 300-750 | 0 | / | ~80 | / | ~10^14^ | 30000 | **13** |
|  | CdSe (7 nm) | 300-650 | 15 | / | ~60 | 8.3×10^3^ | 4.2 × 10^17^ | 270/90000 | **11** |
| SWIR | HgTe CQDs | 350-2430 | 15 | / | 25^4^ | 10 | 1.5×10^11^ | 184/212 | **14** |
|  | HgTe CQDs | 350-2500 | 0 | 0.3@500Hz | 56 | 0.72 | 3.6 × 10^11^ | 0.579 | **15** |
| B | Poly-TPD:F8T2 | 350-500 | 0 | 0.07**^d^** | 3.5 | 0.012 | 4.2×10^10^ | 54.6/118.4 | **Our work** |
| G | D18:PDNBO | 350-630 | 0 | 0.09**^d^** | 22 | 0.1 | 1.1×10^11^ | 72.7/99.5 |  |
| R | PTB7-Th:PZ1 | 350-780 | 0 | 0.09**^d^** | 35 | 0.2 | 3.7×10^11^ | 66.2/242.2 |  |
| SWIR | HgTe CQDs | 350-2350 | 0 | 0.67**^d^** | 72.3 | 0.83 | 2.91×10^11^ | 13/5 |  |

***a.*** *Shot Noise;* ***b****. White Noise;* ***c****. Blackbody Radiation EQE;* ***d****. RMS Noise (1 Hz–1 kHz)*

**Table S7**. A summary of the multispectral FPA properties.

| **Materials** | **Resolution** | **Number of spectral bands** | **Spectral Distribution** | **Dark current** | **Dead pixel rate** | **Reference** |
| --- | --- | --- | --- | --- | --- | --- |
| PeQDs / Si /CQD | 320 × 256 | 3 | UV/Vis/SWIR | / | 0 | **5** |
| CQDs | 10×10 | 4 | R/G/B/IR | / | / | **6** |
| Organic | 10×10 | 3 | R/G/B | / | / | **8** |
| Metamaterials | 10×10 | 3 | 2.5/3.4/4.3 THz | / | / | **16** |
| InAs/GaAs QD | 320 × 256 | voltage-tunable | SWIR-LWIR | / | / | **17** |
| Organic/CQDs | 640×512 | 4 | R/G/B/SWIR | 0.19 pA | ＜1% | **Our work** |

**Reference:**

1. Hu, J., Shen, L. & Sun, G. in 2018 IEEE/CVF Conference on Computer Vision and Pattern Recognition 7132-7141 (2018).

2. Woo, S., Park, J., Lee, J.-Y. & Kweon, I.S. in Computer Vision – ECCV 2018. (eds. V. Ferrari, M. Hebert, C. Sminchisescu & Y. Weiss) 3-19 (Springer International Publishing, Cham; 2018)

3. Michito S et al. Dark current measurement of Type-II superlattice infrared focal plane array detector," *Proc. SPIE 9070,* **9070,** 907015 (2014).

4. Zhang, S. et al. Wafer-scale fabrication of CMOS-compatible trapping-mode infrared imagers with colloidal quantum dots. *ACS Photonics* **10**, 673-682 (2023).

5. Yun, Y. et al. A wide bandgap halide perovskite based self-powered blue photodetector with 84.9% of external quantum efficiency. *Adv. Mater.* **34**, 2206932 (2022).

6. Wu, C. et al. Highly efficient and stable self-powered ultraviolet and deep-blue photodetector based on cs2agbibr6/sno2 heterojunction. *Adv. Opt. Mater.* **6**, 1800811 (2018).

7. Zhang, T. et al. High-performance filterless blue narrowband organic photodetectors. *Adv. Funct. Mater.* **34**, 2308719 (2024).

8. Kim, J. et al. Exciton-scissoring perfluoroarenes trigger photomultiplication in full color organic image sensors. *Adv. Mater.* **35**, 2302786 (2023).

9. Ma, F. et al. High-sensitivity green photodetectors using subphthalocyanine derivatives as photoactive donors. *Phys. Chem. C.* **126**, 13496-13504 (2022).

10. Lim, S.-J. et al. Organic-on-silicon complementary metal–oxide–semiconductor colour image sensors. *Sci. Rep.* **5**, 7708 (2015).

11. Kim, J. et al. A skin-like two-dimensionally pixelized full-color quantum dot photodetector. *Sci. Adv.* **5**, eaax8801.

12. Qiao, J.-W. et al. Efficient ultrathin self-powered organic photodetector with reduced exciton binding energy and auxiliary föster resonance energy transfer processes. *Adv. Funct. Mater.* **33**, 2301433 (2023).

13. Dou, L. et al. Solution-processed hybrid perovskite photodetectors with high detectivity. *Nat. Commun.* **5**, 5404 (2014).

14. Qin, T. et al. Mercury telluride colloidal quantum-dot focal plane array with planar p-n junctions enabled by in situ electric field–activated doping. *Sci. Adv.* **9**, eadg7827.

15. Hu, H. et al. Double-heterojunction-based hgte colloidal quantum dot imagers. *ACS Nano* **19**, 8974-8984 (2025).

16. Zhou, Z. et al. Multicolor t-ray imaging using multispectral metamaterials. *Adv. Sci.* **5**, 1700982 (2018).

17. Vaillancourt, J. et al. A voltage-tunable multispectral 320 × 256 inas/gaas quantum-dot infrared focal plane array. *Semicond. Sci. Tech.* **24**, 045008 (2009).
